# Liquid–liquid phase transition in deeply supercooled Stillinger–Weber silicon

**DOI:** 10.1093/pnasnexus/pgac204

**Published:** 2022-09-23

**Authors:** Yagyik Goswami, Srikanth Sastry

**Affiliations:** Theoretical Sciences Unit and School of Advanced Materials, Jawaharlal Nehru Centre for Advanced Scientific Research, Rachenahalli Lake Road, Bengaluru 560064, India; Theoretical Sciences Unit and School of Advanced Materials, Jawaharlal Nehru Centre for Advanced Scientific Research, Rachenahalli Lake Road, Bengaluru 560064, India

**Keywords:** liquid–liquid phase transition, supercooled liquids, Stillinger–Weber silicon, network-forming liquids, free-energy

## Abstract

The existence of a phase transition between two distinct liquid phases in single-component network-forming liquids (e.g. water, silica, silicon) has elicited considerable scientific interest. The challenge, both for experiments and simulations, is that the liquid–liquid phase transition (LLPT) occurs under deeply supercooled conditions, where crystallization occurs very rapidly. Thus, early evidence from numerical equation of state studies was challenged with the argument that slow spontaneous crystallization had been misinterpreted as evidence of a second liquid state. Rigorous free-energy calculations have subsequently confirmed the existence of a LLPT in some models of water, and exciting new experimental evidence has since supported these computational results. Similar results have so far not been found for silicon. Here, we present results from free-energy calculations performed for silicon modeled with the classical, empirical Stillinger-Weber–potential. Through a careful study employing state-of-the-art constrained simulation protocols and numerous checks for thermodynamic consistency, we find that there are two distinct metastable liquid states and a phase transition. Our results resolve a long-standing debate concerning the existence of a liquid–liquid transition in supercooled liquid silicon and address key questions regarding the nature of the phase transition and the associated critical point.

Significance Statement The debate around the existence of a phase transition between distinct liquid phases in single-component liquids has elicited considerable scientific interest in recent years. The most recent evidence from investigations of water, both computational and experimental, strongly suggests the existence of a liquid–liquid phase transition (LLPT). However, similar results have not been available for silicon, which has been argued to be an extreme case in the family of network-forming liquids that may exhibit a liquid–liquid transition. The phase transition is expected to occur at very deeply supercooled conditions, presenting methodological challenges which we address. Our demonstration of the LLPT in model liquid silicon represents a definitive step in establishing the generality of liquid–liquid transitions in network forming liquids.

## Introduction

The possibility of a phase transition between distinct liquid states in a single-component liquid has been the subject of intense scientific investigation and debate (1). Liquid silicon, which we study here, is one such case where a number of experimental and computational studies have addressed the existence of such a liquid–liquid phase transition (LLPT) (2–6). The most prominent example of a liquid undergoing a LLPT is water. The possibility of a liquid–liquid transition in water was first discussed while attempting to understand the apparent divergence of isothermal compressibility and other quantities in the supercooled state (7,8). Based on molecular dynamics simulations for the ST2 model of water, Poole et al. (9) proposed the existence of a liquid–liquid critical point under metastable conditions. Other scenarios have also been proposed, including those not invoking any singular behavior (10). The possibility of a liquid–liquid transition has since been explored in a large variety of substances, and efforts made to understand its origins (1,11). Verifying the existence of a LLPT experimentally has proved to be immensely challenging both for water (12–14) and for silicon (5, 15). A number of numerical studies have reported the existence of a LLPT in silicon, both using first-principles or *ab initio* methods (6) and using molecular dynamics with a classical empirical potential (3,4). However, the body of numerical evidence pointing to a LLPT was brought into question by the work of Limmer and Chandler (16,17), who argued that the appearance of a second liquid phase was due to the misinterpretation of slow and spontaneous crystallization. Their study included results on models of water, including the ST2 model, as well as silicon modeled by the classical, empirical Stillinger–Weber (SW) potential (18). The debate around water has since been resolved and the existence of the LLPT confirmed in a comprehensive study of the free-energy surface for the ST2 potential by Palmer et al. (19) and for other realistic potentials (20). Equally compelling results have also been reported for models of silica (21,22). For SW silicon, the question has remained open, with recent investigations with free-energy calculations similar to those performed by Limmer and Chandler and Palmer et al. showing only one liquid state before the free-energy barrier with respect to crystallization vanishes at conditions where the phase transition is expected (23). This scenario of spontaneous, barrier-less, crystallization in silicon has since been ruled out in the work of Goswami et al. (24). In that work, the choice of a global order parameter *Q*_6_—typically used in such contexts—to constrain the system to reversibly sample states with different degrees of crystallinity was demonstrated to give misleading results [and we believe, as supported by free-energy calculations in ref. (25), that similar considerations would also apply to the mW model, another system for which spontaneous crystallisation has been argued to rule out the liquid–liquid transition (26)]. However, the question of whether there are in fact two metastable liquid states remains an open and challenging one. Starr and Sciortino (27) set out to understand the relative propensity of different model network forming liquids to display either a stable or metastable LLPT based on the angular rigidity of the tetrahedral bonds. This analysis led to the successful design of a patchy colloidal model that exhibits a LLPT without the intervention of crystallization (28). The analysis in ref. (27) revealed that the angular rigidity for SW silicon was the highest among the models considered. Thus, any phase transition between two metastable liquids would be expected in the deeply metastable regime, possibly prevented by the onset of spontaneous crystallization. Silicon thus assumes special significance among the class of network-forming liquids that have been investigated.

Here, we present results from numerical free-energy calculations for SW silicon at conditions where the possibility of a LLPT is discussed. We use a combination of a constrained sampling protocol and an appropriate order parameter, which has been demonstrated to accurately determine the free-energy barriers to crystallization at deep supercooling (24,29). We develop an extension to that methodology to effectively sample both the liquid state(s) and the transition state with respect to crystallization. Our results show clear evidence of the coexistence between two metastable liquid states, with the characteristic double-well feature in the free-energy surface of the liquid state at the relevant conditions. Through rigorous checks for thermodynamic consistency, including simulations at larger system sizes, we are able to demonstrate that the free-energy reconstructions are robust and consistent with expectations concerning a LLPT (19). We further investigate the behavior of the relevant order parameter (30) and characterize the associated critical fluctuations (20), which behave in accordance with the 3D Ising universality class.

We perform umbrella sampling Monte Carlo (31) (USMC) simulations of silicon modeled using the 3-body SW potential (18) in the constant pressure, temperature, and particle number (NPT) ensemble. The density and the size of the largest crystalline cluster are simultaneously constrained with a harmonic umbrella bias and with a hard-wall bias (29), respectively. Crystalline atoms and clusters of connected crystalline atoms are identified using the local analogue of the Steinhardt–Nelson bond orientational order parameters (32) using the procedure described in refs. (33–35) with cut-offs specific to SW silicon as used in ref. (24). Parallel tempering swaps are performed between adjacent bias windows and adjacent temperatures to enhance sampling of different densities (see the “Materials and methods” section and Supplementary Material for details). The convergence of the simulations is checked by monitoring the decay of the auto-correlation functions of the density and global *Q*_6_ (32). Further, visit and excursion statistics from the parallel tempering swaps are also monitored to determine the efficacy of sampling. Free-energy estimates from the different bias windows and simulation conditions are reweighted using an in-house implementation of the weighted histogram analysis method (20,36) to both obtain unbiased free-energy estimates and reweight across temperature and pressure. Errors are obtained by estimating the number of decorrelated samples based on the integrated autocorrelation time for the slowly relaxing variables, density, and *Q*_6_ (19,36,37). Finally, this reweighting procedure is used to compare directly obtained free-energy profiles with those obtained by reweighting from other conditions, giving identical results (see Supplementary Material). This is a strong indication of converged, equilibrium sampling.

## Free-energy reconstruction from constrained sampling

**Fig. 1.**
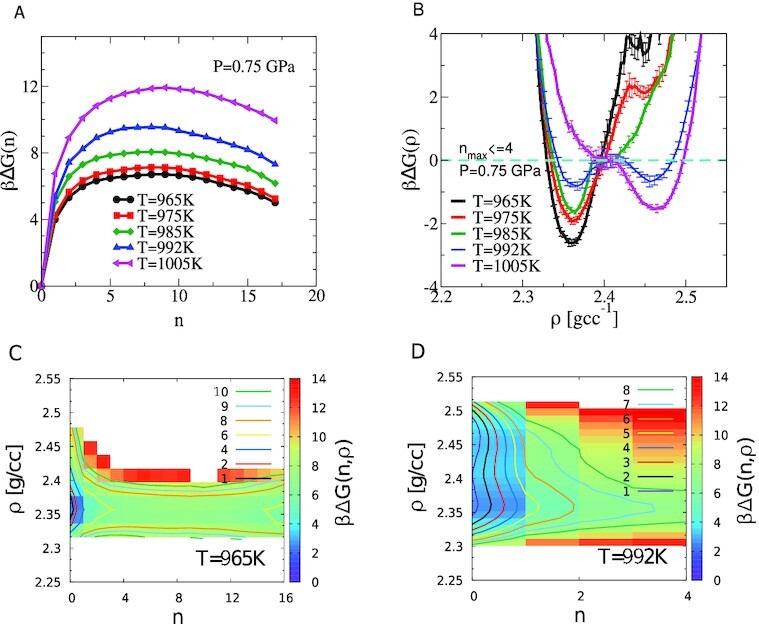
Panel (A) shows the free-energy barrier to crystal nucleation from NPT USMC simulations of *N* = 512 atoms at *P* = 0.75 GPa at the temperatures shown. The free-energy is obtained from the full cluster size distribution using βΔ*G*(*n*) = −*ln*(*P*(*n*)) + const. with the condition βΔ*G*(0) = 0 fixing the value of the constant. The free-energy barrier is finite at all temperatures, with the height at the lowest temperature being 6 to 7* k_B_T*. Panel (B) shows the free-energy as a function of density, obtained from the negative log of the contracted distribution defined in Eq. (1), from the same simulations. Errors are obtained from estimates of the number of decorrelated samples in each constrained simulation. The double well feature at $T=992\, \mathrm{K}$ is indicative of coexistence of the LDL (low-density state) and the HDL (high-density state). Panel (C) shows the free-energy from the joint distribution of density and cluster size at $T=965\, \mathrm{K}$, *P* = 0.75 GPa. The liquid has a low density of $2.37 \, \mathrm{g cc}^{-1}$ even when the degree of crystallinity is zero. Contour lines are placed at the values mentioned in the legend. Panel (D) shows a two order parameter free-energy reconstruction zoomed into the low *n* region, showing a bi-modal feature along density.

Free-energies are reconstructed, in the first instance, for systems of *N* = 512 atoms along the *P* = 0.75 GPa isobar. The size of the largest crystalline cluster, *n*_max_, is constrained within overlapping hardwall constraints [*n*^*lo*^, *n*^*hi*^], while the density is constrained with harmonic bias potentials. Parallel tempering swaps are performed between adjacent windows in *n*_max_, density, and temperature. Total simulation run lengths are in excess of 10^8^ MC steps for each case (at all system sizes) and are compared to the autocorrelation time, number of swaps performed along each axis, and the mean duration required for parallel tempering swaps to “return” to the initial window ( see Supplementary Material Figures S3 and S4). In Figure 1(A), we show the free-energy barrier to crystallization [obtained from the full cluster size distribution, *P*(*n*)] with a finite free-energy cost to the formation of the critical nucleus at each of the temperatures considered.

The choice of temperatures is based on estimates of the LLPT line reported in  (4), where the estimated transition temperature for *P* = 0.75 GPa is *T*  ∼990 to 995 K. We then construct the density distribution subject to the constraint *n*_max_ ≤ 4, integrating over the multivariate distribution to get

(1) \begin{equation*} P(\rho ) = \sum \limits _{\mathit{ n}_{max}=0}^{\mathit{ n}_{max}=4} P(\mathit{ n}_{max},\rho ). \end{equation*}

The corresponding free energies obtained from βΔ*G*(*ρ*) = −*ln*(*P*(*ρ*)) are shown in Figure 1(B), displaying a jump in the most probable density of the liquid across$\mathit{ T}=992\, \mathrm{K}$, and a double-well form at $\mathit{ T}=992\, \mathrm{K}$, indicative of coexistence between two liquids. In Figures 1(C and D), we present two order parameter free-energy reconstructions as a function of the size of crystalline clusters along *x* and the density along *y*. The liquid state(s) can be observed by considering the small *n* region, while the transition state [critical cluster for which βΔ*G*(*n*) is maximum] and the beginnings of the globally stable crystalline basin are observed by scanning along the *x* axis. In these reconstructions, we compute the free-energy from the relative probability of observing a cluster of size *n* in the liquid at density *ρ* (see the “Materials and methods” section and Supplementary Material for more details). Figure 1(C) shows the free-energy at the lowest temperature considered, *T* = 965 K, where the metastable liquid is purely in the LDL, with a barrier with respect to the growth of crystalline order centered at *n* = 8. In Figure 1(D), for *T* = 992 K, two basins are visible at HDL and LDL densities, respectively, in the low *n* region. Integrating over *n* (or *n*_max_) will yield the contracted surface, i.e. the projection of the two-order parameter free-energy along the *ρ* axis shown in Figure 1(B).

## Free-energy reconstructions at larger system sizes

**Fig. 2.**
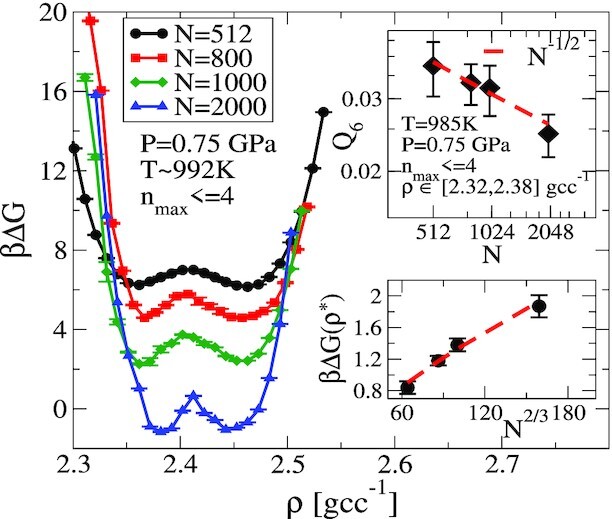
βΔ*G*(*ρ*) from USMC simulations at $T=992\, \mathrm{K}$ at *N* = 512, 800, 1000, and 2000 atoms. Density is sampled subject to the constraint on *n*_max_. error bars are a measure of the number of uncorrelated samples obtained for the free-energy calculation (see Supplementary Material). The bottom inset shows the height of the barrier as a function of system size. The barrier height scales as *N*^2/3^, as expected when a stable interface can form between two phases. Error bars indicate the uncertainty arising from measurements at the bottom of the well and the top of the barrier and the variation in depth of the LDL basin and HDL basin. The top inset shows the scaling of average *Q*_6_ with system size when measured in the low-density basin. The value of *Q*_6_ decreases with *N* as *N*^−1/2^, demonstrating that the low-density phase is disordered. The error bars are the standard deviation obtained from the same samples.

In Figure 2, we show the free-energy profile as a function of density along the *P* = 0.75 GPa isobar at different system sizes ranging from *N* = 512 to *N* = 2000. Convergence is monitored as discussed in the “Materials and methods” section (see FSupplementary Matreial igures S17, S18, and S19). Free-energy estimates at exact coexistence conditions are obtained by reweighting from the available data directly simulated at *P* = 0.75 GPa (see Supplementary Material). We note a slight shift (of $\lt 3 \, \mathrm{K}$) to higher temperatures for the coexistence conditions at larger system sizes, as also noted in (30). The coexistence temperature mentioned in Figure 2 is for *N* = 512.

The formation of a stable interface between two liquid phases will result in a scaling of the barrier height with *N* as *N*^2/3^ (19,38). Results shown in the bottom inset in Figure 2 are consistent with this scaling with system size. Additionally, for the low-density phase to be a disordered phase, the degree of global orientational ordering should scale as *N*^−1/2^ (17,19). We find this to be the case from inspection of the top inset of Figure 2, where the error bars are obtained from the SD of *Q*_6_ measured under the conditions and constraints specified (see also Supplementary Material Figure S20).

## Trends across other state points—absence of bi-modality beyond the critical point

**Fig. 3.**
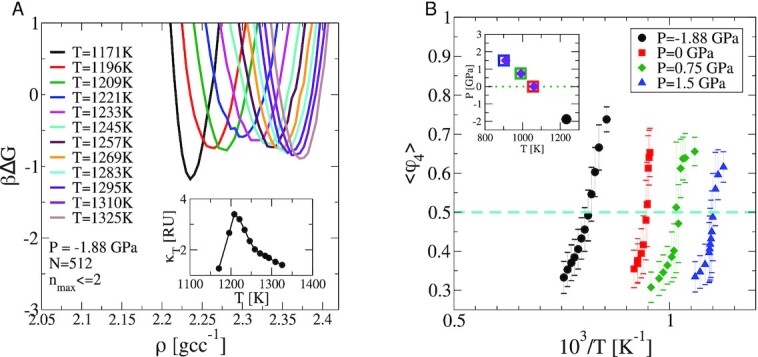
Panel (A) equilibrium sampled density distributions from USMC simulations of *N* = 512 atoms along the *P* = −1.88 GPa isobar. The distributions are unimodal throughout and show no hint of phase separation. *(Inset)*the compressibility measured for different temperatures along the *P* = −1.88 GPa isobar showing a peak at $T\sim 1230\, \mathrm{K}$, generally consistent with that reported by Vasisht et al. (4). Panel (B) The mean fraction of 4-coordinated atoms from the equilibrium sampling probability measured subject to the constraint, *n*_max_ ≤ 4 shown for 3 isobars below the critical point from NPT USMC simulations of *N* = 512 atoms. ϕ_4_ is ∼0.65 at $T=965\, \mathrm{K}$, *P* = 0.75 GPa. Error bars represent the standard deviation of ϕ_4_. (*Inset)* The LLPT line obtained by estimating the point of crossing 〈ϕ_4_〉 = 0.5 for each isobar, shown with symbols of the corresponding color. The LLPT line obtained from βΔ*G*(*ρ*) along each of the 3 isobars, *P* = 0 GPa,   0.75,  and 1.5 GPa (see Supplementary Material for data at *P* = 0  and 1.5 GPa), is shown with violet diamonds. Estimates are found to be consistent with each other and with the equation of state data reported in   (4).

We perform USMC simulations constraining only the largest cluster size with a hardwall bias at conditions far from coexistence, where a single liquid phase exists. A set of overlapping bias windows is used to constrain *n*_max_ and the density distribution is measured subject to a constraint of *n*_max_  ≤  4. Parallel tempering swaps across temperatures enhance the sampling of different values of density and convergence is monitored (see Supplementary Material, Figures S8, S9, S10, and S11). Away from coexistence this procedure gives quantitatively similar estimates of βΔ*G*(*ρ*) as the procedure where both *n*_max_ and *ρ* are constrained (see Supplementary Material Figure S12 and Supplementary Material text for details). We perform similar USMC computations (both variants) along the *P* = 0 GPa and *P* = 1.5 GPa isobars for a range of temperatures straddling the LLPT (see Supplementary Material Figures S13, S14, S15, and S16), as well as at a negative pressure of *P* = −1.88 GPa, which is in the supercritical region of the phase diagram reported in  ref. (4). In this region, the extension of the LLPT line corresponds to a locus of maximum compressibility, also known as the Widom line (4,39). No phase separation is expected to occur, though weak bi-modality in the density distribution may be observed at small system sizes when measured in close proximity to the critical point. One finds no indication of a double-well feature in the free-energy reconstructions along the *P*= −1.88 GPa isobar shown in Figure 3(A) suggesting a fully continuous change in the character of the liquid across the Widom line. From the equilibrium sampling distribution of the fraction of 4-coordinated (LDL-like) atoms, ϕ_4_ (see Supplementary Material), we extract the mean and standard deviation and plot 〈ϕ_4_〉 as a function of temperature along isobars, as shown in Figure 3(B). At coexistence conditions, the liquid is composed of equal fractions of LDL-like and HDL-like atoms, enabling an estimate of the LLPT temperature across which the fraction of 4-coordinated atoms changes sharply, with larger fluctuations around a mean of 0.5 in the vicinity of the transition temperature, as discussed in (40). The change in the fraction is more gradual across the *P* = −1.88 GPa isobar, indicative of a continuous transformation in the properties of the liquid as seen in Figure 3(A).

## Energy and density dependent distributions and critical behavior

**Fig. 4.**
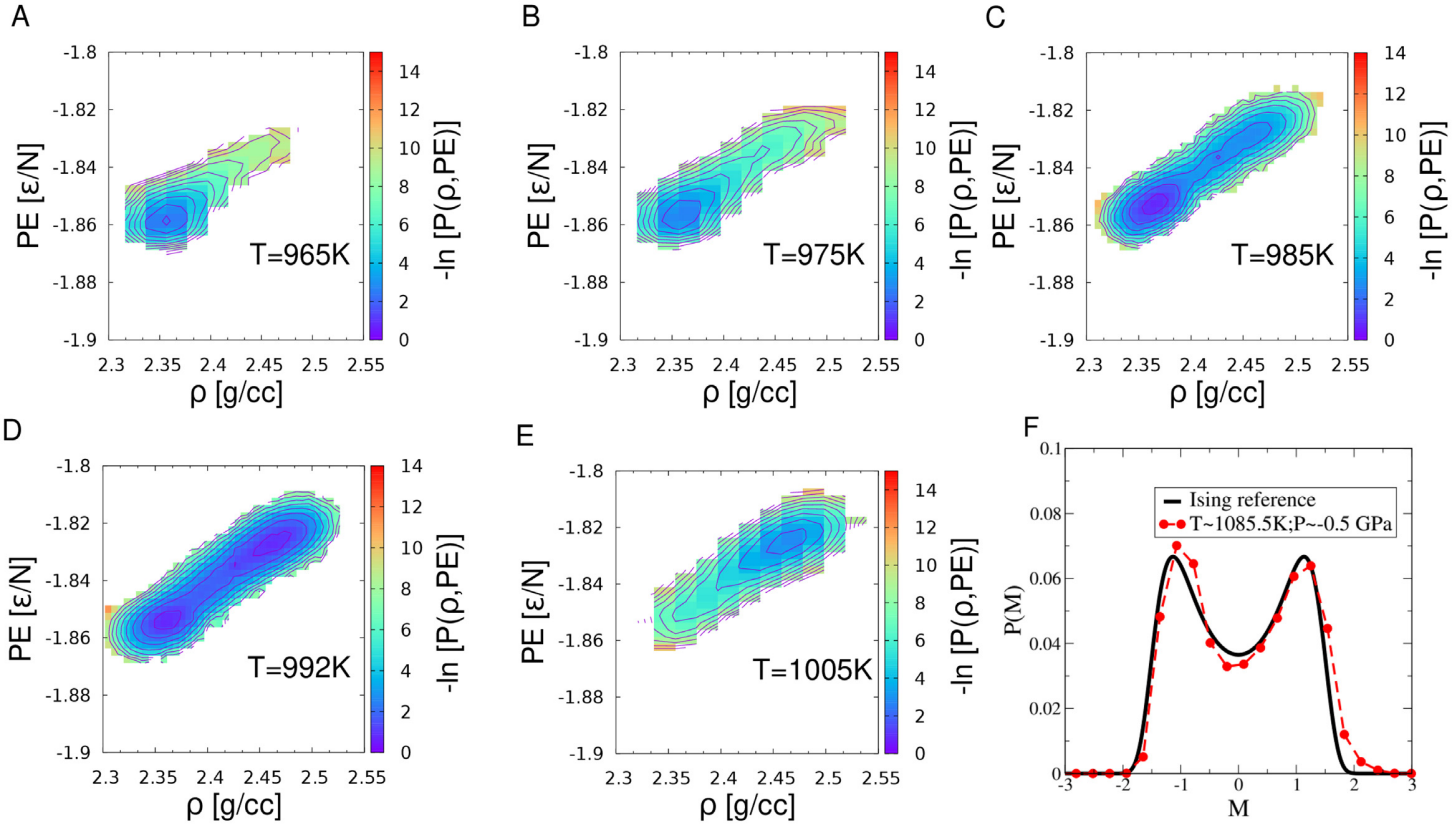
Negative log of the distribution of density and potential energy per atom obtained subject to the constraint of *n*_max_ ≤ 4 along the *P* = 0.75 GPa isobar at five temperatures, $T=965\, {\rm K}$ (A), $T=975\, {\rm K}$ (B), $T=985\, {\rm K}$ (C), $T=992\, {\rm K}$ (D), and $T=1005\, {\rm K}$ (E). Data are obtained from NPT USMC simulations of *N* = 512 atoms. The two liquid phases differ both energetically and in density. Panel (F) shows a comparison of the distribution of the field-mixing order parameter with the reference Ising 3D distribution at the critical point. The critical point and the field mixing coefficient, *s*, are estimated by iteratively reweighting free-energy estimates, obtained directly from umbrella sampling simulations at *P* = 0 GPa, to different *T, P*, and minimising the difference between *P*(*M*) and *P_ising_*(*M*) (see Supplementary Material), where *M* = *ρ*+ *sE* is rescaled to have unit variance. The estimate of the critical point obtained from this procedure is $\mathit{ T}_c=1085.5\, {\rm K},~\mathit{ P}_c=-0.5~{\rm GPa}$.

In Figure 4, we show the multivariate distribution of density and potential energy per atom, subject to the constraint of *n*_max_  ≤ 4 as in Figure 1(B). Here, one observes basins corresponding to the two liquids, a high energy–high-density liquid and a low energy–low-density liquid, with a double-well at *T* = 992 K as in Figure 1(B). The fact that the HDL has a higher energy and is more stable at the high temperature side of the transition suggests that the LDL has a lower entropy or fewer favorable configurations. This behavior is consistent with expectations derived from using the Clausius–Clapeyron equation (*dP*/*dT*)_*LLPT*_ = Δ*S*/Δ*V*, which relates the slope of the transition line to the difference in entropy and density between the two liquid phases (41). The implication of a negative slope for the transition line is that the lower density (higher volume) phase has a lower entropy (42).

Recent work has investigated the nature of critical fluctuations associated with the LLPT in water, which is generally understood to be of the 3D Ising universality class (20). The critical order parameter has been shown to be a linear combination of the density and potential energy (30) (*M* = *ρ* + *sE*) and its probability distribution at the critical point can be well represented by a standard form (43) (see Eq. 4 in the “Materials and methods” section). In order to identify the field-mixing parameter, *s*, and the critical point, we follow a procedure of iteratively estimating *T_c_*, *P_c_* (to which the histograms are reweighted), and *s* for which the order parameter distribution best matches the reference distribution (see the “Materials and methods” section, Supplementary Material text, and Figure S21), following the approach of Debenedetti et al. (20). Figure 4(F) shows the results of this procedure with the estimated critical point and a value of *s* = 0.6, which indicates that the distribution of the order parameter agrees closely with the expectation for the 3D Ising universality class. The critical parameters reported in (4) (*T_c_* = 1120* K*,  *P_c_* = −0.6 GPa) are in reasonable agreement with the values we obtain in the present analysis.

## Comparison with analyses for other models

Free-energy investigations of the LLPT for similar tetrahedrally ordered liquids have shown typical barriers of ∼1 to 4 *k_B_T*. While the barrier height increases with system size, performing constrained simulations at arbitrarily large system sizes is prohibitively expensive. For ST2 water, Palmer et al. (19) report a barrier height of ∼4 *k_B_T* with *N* = 192 molecules at conditions of coexistence far from the critical point. Poole et al. (44) have reported similar barrier heights earlier with *N* = 216 molecules. Simulations of silica by Chen et al. (21) have identified a barrier of less than 4 *k_B_T* away from the critical point, but for a system size of *N* = 1500 atoms. Recent work with two variants of the TIP4P model (which also report analysis of critical fluctuations as belonging to the Ising universality class) has reported density histograms corresponding to a barrier height of less than 2  *k_B_T* for *N* = 300 (20). The barrier heights we obtain of 0.8 k_B_T for *N* = 512 and 1.9 k_B_T for *N* = 2000 are thus comparable to these earlier reported values.

## Discussion

In summary, we find through rigorous free-energy calculations and extensive analysis of the consistency of our results, that two well-defined metastable liquid states, with corresponding free-energy minima, exist in supercooled SW silicon. Coexistence conditions are identified in the subcritical part of the phase diagram that are in agreement with estimates reported previously from equation of state studies. At each of the state points considered, a clear and significant free-energy barrier to crystal nucleation is observed, ruling out the possibility that the low-density liquid is a transient artifact resulting from slow, spontaneous crystallization. At several state points [in particular $\mathit{ T} = 965 \, \mathrm{K}$, *P* = 0.75 GPa shown in Figure 1(C)] we observe a free-energy minimum corresponding to the low-density liquid phase, with a large fraction of tetrahedrally coordinated atoms and zero crystallinity, decisively ruling out the slow crystallization scenario. The free-energy barrier between the two liquids is found to scale with the size of the simulated system—an important test of the presence of a first-order transition. Reweighting of free-energy profiles across conditions results in identical results, providing a strong test of converged equilibrium sampling. We also find that the same analysis finds no evidence of phase separation when performed along an isobar in the super-critical region of the phase diagram, also consistent with the two-critical point scenario (4,9). We note that the density difference between the two liquids is small and remains small as distance from the critical point increases, in contrast to the case of other similar network-forming liquids such as ST2 water (19,44) and WAC silica (21,22). Given the low barriers to crystallization for silicon under these conditions and the small difference in the densities of the two liquid phases, the barrier separating the two phases is expected to be small. However, the scaling of barrier height with system size shown here confirms the existence of two well-defined metastable liquid phases. The barrier heights are of comparable order to these and to other cases such as ST2 water (19), TIP4P water (20), and silica (21). The two liquid states do not differ in density alone, as shown by a free-energy reconstruction along two order parameters, density and potential energy per atom, subject to the constraint of low *n*_max_. These point to the interplay of energy and entropy in driving the transition, as discussed in the context of other liquids showing a LLPT. Our work thus provides a comprehensive analysis that resolves the long-standing debate concerning the existence of a liquid–liquid transition in supercooled SW silicon. Taken together with the simulation investigations in the case of water and silica, and experimental results concerning water and silicon, there is now a preponderance of evidence in support of LLPTs in pure substances.

## Materials and methods

### Interaction potential and simulation protocol

We use the classical three-body SW potential to model silicon (18). Parametrization is shown in Supplementary Material Table S1. Monte Carlo simulations are performed in the NPT ensemble. Enhanced reversible sampling is achieved by using the umbrella sampling scheme (31). An in-house code with an efficient double-sum implementation (45) of the three-body SW potential was used for the USMC simulations. The bias variables are the size of the largest crystalline cluster, *n*_max_, and the density *ρ*. A hard wall bias (which is zero within prescribed limits and infinite outside) is used to constrain the *n_max_* values as used in refs. (24,29) and a harmonic bias constrains the density. Parallel tempering swaps are performed across temperature, *n*_max_ bias, and density bias windows. In simulations where only *n*_max_ is constrained, far from coexistence conditions, parallel tempering swaps are only performed across temperature and *n*_max_ bias windows. Swaps are performed between adjacent windows in *n*_max_, density and adjacent temperatures every 2 × 10^2^ MC steps, 10^3^ MC steps, and 2 ×   10 ^3^ MC steps, respectively. Convergence is determined by monitoring the decay of the time-autocorrelation functions of the density and of the global bond orientational order parameter, *Q*_6_ (32). These are found to decay in less than *τ*  ∼10^5^ to 10^6^ MC sweeps at all conditions and system sizes considered. Simulations lengths exceed 10^8^ MC steps at all the conditions studied, with histograms sampled over ∼100 to 200τ for each window.

Statistics of traversal due to parallel tempering swaps are also used to determine adequate sampling. More than 10^4^ parallel tempering swaps are performed in each direction, with observed mean return times being ∼10^4^ to 10^6^ MC steps for simulations in each window. The return time is the number of MC steps before a simulation returns to its initial temperature or bias potential after being swapped out as a result of the replica exchanges. Further details on the umbrella sampling and parallel tempering scheme are provided in the Supplementary Material.

### Defining atom types

Bulk crystalline atoms are identified as those with high degree of local tetrahedral ordering and also surrounded by similarly tetrahedrally ordered atoms. We use the cut-offs described in the Supplementary Material and also in (24, 35, 46) (see Supplementary Material Figure S1). The local order is identified by using the local bond orientational order for each atom, *q*_3_(*i*). Neighbouring atoms with correlated neighbourhoods are said to be “bonded”, with the correlation function used being *q*_3_(*i*).*q*_3_(*j*). Atoms bonded to three or more neighbors are defined as bulk crystalline atoms. A 4-coordinated or “LDL” atom is identified as one with high local *q*_3_ but bonded to fewer than three of its neighbors. The fraction of such 4-coordinated liquid-like atoms, ϕ_4_, is also used to estimate coexistence conditions. At coexistence, the fraction of such 4-coordinated atoms in LDL-like local structures is expected to be ∼0.5 (40,42). The details of the cut-offs used and the relevant distributions are shown in the Supplementary Material.

### Free-energy as a function of cluster size and density

We measure the unbiased probability of observing a cluster of size *n* in the liquid at density *ρ* and take the negative log to obtain a free-energy as shown below:

(2) \begin{equation*} \Delta G(n,\rho ) = -k_BT \ln (P(n,\rho )). \end{equation*}

To obtain this, one is required to obtain the following equilibrium probability distribution:

(3) \begin{equation*} P(n,\rho ) = \frac{1}{\tau _{s}}\sum _{t=0}^T\frac{N(n,t)}{N(0,t)}\delta (\rho (t) - \rho ))\quad if\quad n_{max}^l \le n \le n_{max}^u. , \end{equation*}

Sampling is performed in the biased ensemble and we use the iterative scheme of the weighted histogram analysis method (36,37) (described in the following section) to obtain the unweighted, normalised distribution, *P*(*n*, ρ) from which we obtain the free-energy surfaces shown in Figures 1(C) and (D). Note that the contracted free-energy surface in Figures 1(B) and 4(A to E) are obtained by first constructing the unbiased estimate for *P*(*n*_max_, ρ) from free-energy reweighting. Then the contracted free-energy, βΔ*G*(ρ), is obtained by summing *P*(*n*_max_, ρ) up to the chosen largest value of *n*_max_ and taking the negative logarithm (see Eq. 1).

### Reweighting and stitching free-energy surfaces

For umbrella sampling runs with two order parameters, we employ an in-house code that implements the self-consistent iterative scheme of the WHAM equations (36,37) (see Supplementary Material Figure S2 and Supplementary Material text for details). Errors are estimated from the number of decorrelated samples and the integrated autocorrelation times. Tests for thermodynamic consistency are performed by comparing reweighted estimates of the free-energy to directly measured estimates at different conditions (see Supplementary Material Figure S5, S6, and S7). For single order parameter umbrella sampling simulations only the largest cluster size is constrained with parallel tempering across temperatures enhancing sampling of density (see Supplementary Material). The two methods agree quantitatively for conditions far from coexistence, whereas only the full two-order parameter US simulations give reliable results at or near coexistence conditions (see Supplementary Material for details).

### Critical fluctuations of the order parameter

We investigate whether the liquid–liquid critical point belongs to the 3D-Ising universality class by comparing the probability distribution of the relevant order parameter with the reference distribution for the 3D-Ising model. In the case of the Ising model, the magnetisation, *M*, undergoes critical fluctuations in the vicinity of the critical point. In the case of the LLPT, the relevant order parameter is a linear combination of density and the potential energy (*ρ* + *sE*) (30). The following general expression is found to be a good approximation to the distribution of *M* (43)

(4) \begin{equation*} P_{ising}(M) \propto exp \Biggl \lbrace - \left(\frac{M^2}{M_0^2} -1 \right)^2\left( a\frac{M^2}{M_0^2} +c \right)^2 \Biggr \rbrace . \end{equation*}

The appropriate choice of constants yields a distribution of unit variance (see Supplementary Material for details). The distribution of the order parameter, *M* = *ρ* + *sE*, is expected to match the reference distribution at the critical point. The critical point is identified by finding the optimal set of *T_c_*, *P_c_*, *s* that minimizes the root-mean-squared error of *P*(*M*) with respect to *P_ising_*(*M*) (see Supplementary Material for details). This procedure gives both an estimate of the critical point as well as the field-mixing parameter, *s*.

## Supplementary Material

pgac204_Supplemental_Files

## Data Availability

All data are included in the manuscript and/or supplementary Material.

## Preprints

A preprint of this article is published at https://doi.org/10.48550/arXiv.2205.13841
