# Supplementary material for: Liquid–liquid phase transition in deeply supercooled Stillinger–Weber silicon
Source: PNAS Nexus. 2022 Sep 23;1(4):pgac204. doi: 10.1093/pnasnexus/pgac204 (PMC9802493; doi:10.1093/pnasnexus/pgac204)
Supplement: pgac204_Supplemental_Files [file pgac204_supplemental_files.zip › PNASNEXUS-PNASNEXUS-2022-00884-T-s03.pdf]

Supplementary Information for

Liquid-liquid phase transition in deeply supercooled Stillinger-Weber silicon

Yagyik Goswami, Srikanth Sastry

Srikanth Sastry
sastry@jncasr.ac.in

This PDF file includes:

Supplementary text
Figs. S1 to S21
Table S1

The supplementary information contains additional details on the model and methods and several sections with details relevant to the results shown in the main text. These are organised as follows:

1. Additional details of the model and methods.
2. Convergence tests for simulations of $N = 512$ atoms along the $P = 0.75$ GPa isobar.
3. Comparison of free energy reconstruction from umbrella sampling with both n_{max} and ρ biased to umbrella sampling simulations where only n_{max} is biased, far from coexistence conditions.
4. Free energy reconstructions along the $P = 0$ GPa and $P = 1.5$ GPa isobars.
5. Free energy reconstruction at larger system sizes.
6. Fitting order parameter distribution to the Ising universality class.

1 Additional Details of the Model and Methods

We construct the free energy landscape here using two methods, umbrella sampling with a hard wall bias potential and a harmonic bias potential.

Here we describe a prescription to extend these methods to the case of two order parameters and discuss the results obtained.

Model - The Stillinger-Weber potential

The Stillinger-Weber potential consists of a two-body term and a three-body term, U_2 and U_3, respectively. [1]

$$U_{SW} = \sum_{j>1}^{N} U_2(r_{ij}) + \sum_{j>i<k}^{N} U_3(\mathbf{r_i}, \mathbf{r_j}, \mathbf{r_k}) \tag{1}$$

The r_i, r_j, r_k are position vectors for atoms i, j, k. r_{ij} is the distance between the ith and j^{th} atoms. N is the total number of atoms in the system.

$$U_2(r_{ij}) = \begin{cases} \epsilon A \left(\frac{B}{r_{ij}^4} - 1 \right) e^{\frac{1}{r_{ij} - r_c}} & if \quad r < r_c \\ 0 & if \quad r \geq r_c \end{cases} \tag{2}$$

The three-body interaction term is defined by

$$\begin{aligned} U_3(\mathbf{r_i}, \mathbf{r_j}, \mathbf{r_k}) = h(r_{ij}, r_{ik}, \theta_{jik}) + h(r_{ij}, r_{jk}, \theta_{ijk}) + \\ h(r_{ik}, r_{jk}, \theta_{ikj}) \end{aligned} \tag{3}$$

In turn,

$$h(r_{ij}, r_{ik}, \theta_{jik}) = \begin{cases} \epsilon\lambda \left[cos\theta_{jik} + \alpha \right]^2 e^{\frac{\gamma}{r_{ij} - r_c} + \frac{\gamma}{r_{ik} - r_c}} & if \quad r_{ij}, r_{ik} < r_c \\ 0 & if \quad r_{ij} \quad or \quad r_{ik} \geq r_c \end{cases}$$

The constants used in the equations above are listed in Table S1.

Symbol	A	B	r_c	λ	α	γ
Value	7.04955	0.60222	1.80	21.0	1/3	1.20

Table S1: Values of the constants in the Stillinger-Weber potential used to model silicon.

Order Parameters

The bond orientational order parameters of Steinhardt, Nelson and Ronchetti [2] are used to distinguish bulk crystalline atoms from liquid-like atoms and further to distinguish LDL-like liquid atoms from HDL-like atoms. Specifically, the local analogue of this order parameter can be used to distinguish the neighbourhoods of individual atoms and classify them as being ordered or disordered.

$$q_{lm}(i) = \frac{1}{n_b(i)} \sum_{j=1}^{n_b(i)} Y_{lm}[\theta(\mathbf{r_{ij}}), \phi(\mathbf{r_{ij}})] \tag{4}$$

The corresponding order parameter, summed over $m's$ is

$$q_l(i) = \left[\frac{4\pi}{(2l+1)} \sum_{m=-l}^{l} |q_{lm}(i)|^2 \right]^{1/2} \tag{5}$$

Here, we use $q_3(i)$, noting that using $q_6(i)$ is equivalent and results in a very similar classification [3]. The number of neighbours, $n_b(i)$, is taken to be the number of atoms within the first coordination shell of the pair-correlation function, i.e., atoms within a cut-off of 2.95Åfrom the reference atom. Other works have considered other definitions, such as considering only the four nearest neighbours. However, when there are more than four atoms at similar distances from the reference atom, certain artefacts arise such as the apparent decrease of tetrahedral ordering with density or an increase with pressure [3]. We therefore employ a distance-based cut-off to specify nearest neighbours. To identify crystalline atoms, we compute the correlations in the local orientational order of neighbouring atoms, following the prescription described in the literature[4, 5, 6]. Atoms with correlated neighbourhoods of high local orientational order are classified solid-like atoms.

Quantitatively, this correlation is given by the quantity, [5, 7, 8]

$$Re\left(q_3(i).q_3(j)\right) = Re\left(\sum_{-3}^{3} q_{3m}(i) q_{3m}^*(j)\right) \tag{6}$$

An atom i and an atom j are considered to be "bonded" if $Re(q_3(i).q_3(j)) < -0.23$. We note here the significance of the the cut-off value of -0.23 which demands that the crystal structure formed is diamond cubic, to the exclusion of the hexagonal crystal structure which also has local tetrahedral ordering [7, 9]. Crystalline atoms have a $q_3 > 0.6$ and are "bonded" to at least 3 neighbours. Further, crystalline atoms within the SW-cutoff distance of each other belong to the same cluster. In this study we consider both the size of the largest cluster, n_{max} and the full distribution of cluster sizes $P(n)$. We observe that using $q_6(i).q_6(j)$ to identify crystalline atoms gives nearly identical results when the appropriate cut-off is chosen. The choice of cut-off will depend on whether a normalisation factor is included in the definition [10, 9]. LDL-like atoms have a high $q_3(i) > 0.6$, showing high tetrahedral ordering, but have fewer than 3 neighbours with similar ordering. Finally, HDL-like atoms have disordered neighbourhoods with 5 or more neighbours. Fig. S1 shows the distributions of q_3, $Re\left(q_3(i).q_3(j)\right)$ and the number of bonded neighbors for typical crystalline, LDL and HDL configurations.

Umbrella sampling

Umbrella sampling Monte Carlo (USMC) simulations were performed at the state points mentioned with a hard wall bias applied that strictly constrains the size of the largest crystalline cluster to be between n_{max}^l and n_{max}^u as described in Eq. 9. The full cluster size distribution is used to calculate the free energy upto an additive constant using Eq. 7.

$$\beta\Delta G(n) = -\ln[P(n)] + \text{const.} \tag{7}$$

Parallel tempering swaps between adjacent windows are carried out to enhance sampling and speed up equilibration. The general expression for the Hamiltonian under application of bias is given by:

$$H_C = H + W_1(n_{max}) + W_2(\rho) \tag{8}$$

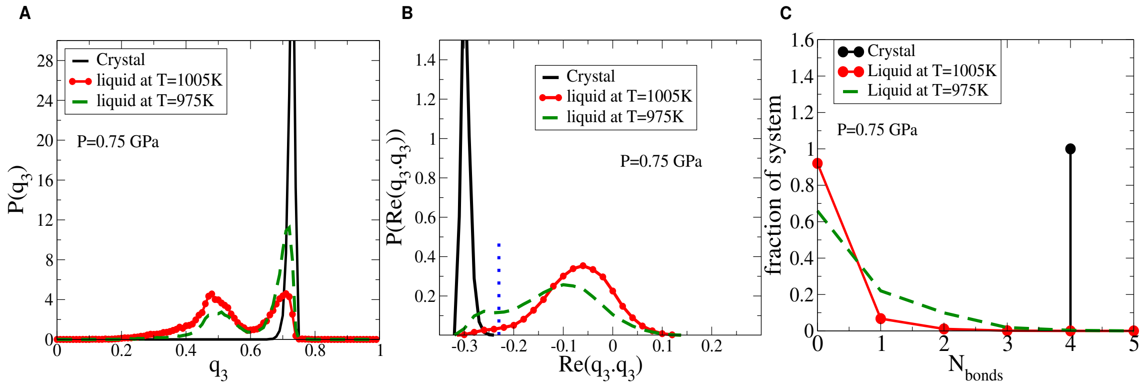

Figure S1: Panel A shows the q_3 distributions for the three types of atoms, panel B shows the distribution of the correlation function and panel C shows the distribution of neighbours that are "bonded" according to the correlation function for the three phases.

where H is the original Hamiltonian, $W_1(n_{max})$ represents the bias potential on n_{max}, and $W_2(\rho)$ is the bias potential on ρ.
Here, W_1 is defined by

$$W_1 = \begin{cases} 0 & n_{lo} \leq n_{max} < n_{hi} \\ \infty & otherwise \end{cases} \tag{9}$$

For W_2, a harmonic bias of the form

$$W_2(\rho; \rho_0, k_\rho) = \frac{1}{2} k_\rho (\rho - \rho_0)^2 \tag{10}$$

is used to enhance sampling around a desired value of ρ, labelled ρ^0. We can write the constrained Hamiltonian as:

$$H_C = H + W_1(n_{max}; n_{lo}, n_{hi}) + W_2(\rho; \rho_0, k_\rho) \tag{11}$$

The unbiased expectation value of some system property such as the density, ρ can be written as (in general for a bias applied on any combination of collective variables such as (n_{max}, ρ)):

$$\langle \rho \rangle = \frac{\left\langle \rho e^{\beta W_1 + W_2} \right\rangle_C}{\left\langle e^{\beta W_1 + W_2)} \right\rangle_C} \tag{12}$$

The expectation subscript C is the sampled probability from the simulation under the modified Hamiltonian. Likewise, W_C is the bias potential that describes the constrained ensemble.

Parallel tempering

The general expression for probability of acceptance of parallel tempering swaps in the NPT ensemble between simulations indexed i and j is given by

$$\begin{aligned} P_{accept} = min\Bigg(1, exp\Big[\big[(E_i - E_j) + P(V_i - V_j)\big](\beta_i - \beta_j)\Big] \\ exp\big[-\beta_j W_i(n_{max_j}) - \beta_i W_j(n_{max_i})\big] \\ exp\big[\beta_i W_i(n_{max_i}) + \beta_j W_j(n_{max_j})\big]\Bigg) \end{aligned} \tag{13}$$

The details of parallel tempering are as follows:

- Consider N independent simulations run in parallel - different temperatures or different bias potentials.
- To ensure better sampling of the configuration space and consequently of the order parameter, we swap adjacent configurations periodically.
- Two types of swaps are performed, one type where simulations with different temperatures but the same bias potential exchange configurations and one type where simulations at the same temperature but different bias potentials exchange configurations.
- A swap between adjacent simulations indexed i and j, at different temperatures, $1/\beta_i$ and $1/\beta_j$, but with the same bias potential is executed with a probability of $min\Bigg(1, exp\big[(E_i - E_j) + P(V_i - V_j)\big](\beta_i - \beta_j)\Bigg)$
- For cases where β is the same but the bias potential varies, the probability is $min\left(1, exp\left[\beta(W_N - W_O)\right]\right)$
- Here, the term $W_N - W_O$ represents the sum of the bias potentials after the swap minus the sum of the bias potentials before the swap (the sum being over the bias applied on the two runs in consideration.

$$\begin{aligned} W_N &= W_j(n_{max_i}) + W_i(n_{max_j}) \\ W_O &= W_i(n_{max_i}) + W_j(n_{max_j}) \end{aligned}$$

In all simulations replica exchanges are attempted across adjacent temperatures and bias windows. Thus, in simulations where both n_{max} and ρ are constrained, parallel tempering swaps are performed across T, $[n^l_{max} : n^u_{max}]$ and ρ_0. For the hard wall bias, the swap is accepted with probability 1 if the n_{max_i} and n_{max_j} are both within the new constraints after the swap and rejected otherwise.

Unbiasing and stitching free energies with WHAM

The weighted histogram analysis scheme[11, 12] is used to unbias and stitch together free energy estimates from the different independent umbrella sampling simulations, as well as to reweigh the unbiased distributions to other values of T, P. We describe the procedure generally, before describing the exact details of implementation in each of the cases where free energy stitching and/or reweighting is performed. In what follows, we begin by considering a general case where our goal is to obtain the unbiased equilibrium distributions of $E, \rho(1/V)$ and any other order parameter(s). In our case, the additional order parameter is n_{max}.

The value of the bias potential $W_i(E, \rho, n_{max})$ in a simulation indexed i refers to the total bias that includes a bias potential on n_{max} and the density ρ (but not on E in our simulations), shown in Eq. 11:

$$W_i(E, \rho, n_{max}) = W_1(n_{max}; n^i_{lo}, n^i_{hi}) + W_2(\rho; \rho^i_0, k^i_\rho), \tag{14}$$

Given R NPT simulations performed under different conditions (different temperature, pressure and/or bias), one obtains equilibrium unbiased estimates of E, ρ, n_{max}, i.e., the internal energy, volume and order parameter which can be reweighted to nearby temperatures and pressures. The density of states, $\Omega(E, \rho, n_{max})$, is given by iteratively solving the following self-consistent equations[11, 12, 13]

$$\begin{aligned} \Omega(E, \rho, n_{max}) &= \frac{\sum\limits_{i=1}^{R} H^b_i(E, \rho, n_{max})}{\sum\limits_{i=1}^{R} N_i e^{-\beta_i E} e^{-\beta_i \frac{P_i N}{\rho}} e^{-\beta W_i(E,\rho,n_{max})} e^{F_i}} \\ e^{-F_i} &= \sum_{\{E,V,n_{max}\}} \Omega(E, \rho, n_{max}) e^{-\beta_i E} e^{-\beta_i \frac{P_i N}{\rho}} e^{-\beta W_i(E,\rho,n_{max})} \end{aligned} \tag{15}$$

where $H_i^b(E, \rho, n_{max})$ is the histogram obtained in simulation i, N_i is the total number of entries from simulation i, and F_i are the shifts applied to each window. These equations are solved iteratively to self-consistency to obtain the shifts corresponding to each simulation window and the density of states, $\Omega(E, \rho, n_{max})$.

On-the-fly unbiasing, i.e., factoring out the Boltzmann weight associated with the bias potential for each configuration sampled, is useful to obtain the unbiased distributions of all quantities, under the given conditions of P, β.

For this, the histograms for each simulation of length τ_i are computed as

$$H_i(E, \rho, n_{max}) = \frac{1}{\tau_i} \sum_{t=1}^{\tau_i} \delta(E(t) - E)\delta(\rho(t) - \rho)\delta(n_{max}(t) - n)e^{\beta_i W_i(E,\rho,n_{max})}, \tag{16}$$

as opposed to a flat histogram count without the inverse of the Boltzmann factor for the bias potential. The iterative equations employing H_i are:

$$\begin{aligned} \Omega(E, \rho, n_{max}) &= \frac{\sum_{i=1}^{R} H_i(E, \rho, n_{max})}{\sum_{i=1}^{R} N_i e^{-\beta_i E} e^{-\beta_i \frac{P_i N}{\rho}} e^{F_i}} \\ e^{-F_i} &= \sum_{\{E,V,n_{max}\}} \Omega(E, \rho, n_{max}) e^{-\beta_i E} e^{-\beta_i \frac{P_i N}{\rho}}. \end{aligned} \tag{17}$$

We can write an unnormalised distribution from the density of states, reweighted to some target β, P as:

$$\begin{aligned} N_{ub}(E, \rho, n_{max}\beta, P) &= \Omega(E, \rho, n_{max}) e^{-\beta E} e^{-\beta \frac{PN}{\rho}} \\ N_{ub}(E, \rho, n_{max}; \beta, P) &= e^{-\beta E} e^{-\beta \frac{PN}{\rho}} \frac{\sum_{i=1}^{R} H_i(E, \rho, n_{max})}{\sum_{i=1}^{R} N_i e^{-\beta_i E} e^{-\beta_i \frac{P_i N}{\rho}} e^{F_i}} \end{aligned} \tag{18}$$

This can be explicitly normalised to obtain the probability distributions,

$$P_{ub}(E, \rho, n_{max}; \beta, P) = \frac{\Omega(E, \rho, n_{max}) e^{-\beta E} e^{-\beta \frac{PN}{\rho}}}{\sum_{\{E,\rho,n_{max}\}} \Omega(E, \rho, n_{max}) e^{-\beta E} e^{-\beta \frac{PN}{\rho}}} \tag{19}$$

which are written in terms of H_i as

$$\begin{aligned} P_{ub}(E, \rho, n_{max}; \beta, P) &= \frac{\sum_{i=1}^{R} H_i(E, \rho, n_{max})}{\sum_{i=1}^{R} N_i e^{(\beta-\beta_i)E} e^{(\beta P - \beta_i P_i)\frac{N}{\rho}} e^{F_i}} \\ e^{-F_i} &= \sum_{\{E,\rho,n_{max}\}} P_{ub}(E, \rho, n_{max}) e^{(\beta-\beta_i)E} e^{(\beta P - \beta_i P_i)\frac{N}{\rho}} \end{aligned} \tag{20}$$

from which we can obtain the free energies. The procedure is as follows:

1. The F_i are initialised to arbitrary non-zero values.
2. $P_{ub}(E, \rho, n; \beta, P)$ in Eq. 20 (a) is computed using histograms H_i.
3. F_i are computed from $P_{ub}(E, \rho, n; \beta, P)$ using Eq. 20 (b)

4. Steps 2 and 3 are repeated till a tolerance value of 10^{-4} is reached for the quantity, $\sqrt{\frac{1}{R}\sum\limits_{i=1}^{R}(F_i - F_i^{old})^2}$.

Integrated auto-correlation time and errors are computed from the auto-correlation function of the order parameter (we have used ρ),

$$C_\rho(t) = \frac{\langle \rho(t)\rho(0)\rangle - \langle\rho\rangle^2}{\langle\rho^2\rangle - \langle\rho\rangle^2} \tag{21}$$

The integrated auto-correlation time is obtained from the self auto-correlation as

$$g = 1 + 2\sum_{t=1}^{T-1}\left(1 - \frac{t}{T}\right) C_\rho(t). \tag{22}$$

We desire the integrated auto-correlation time for the slowly varying density, ρ, and the resultant measure of the error in our estimates of $\beta\Delta G(\rho)$. We thus weight sampling according to the number of decorrelated samples obtained as a function of ρ. The error as a function of ρ is

$$\omega(\rho) = \left(\sum_{i=1}^{N_{sim}} \frac{g_i^\rho}{\langle H_i(\rho)\rangle}\right)^{1/2} \tag{23}$$

The WHAM equations are then modified as:

$$\begin{aligned} P_{ub}(E,\rho,n_{max};\beta,P) &= \frac{\sum\limits_{i=1}^{R} g_i^{-1} H_i(E,\rho,n_{max})}{\sum\limits_{i=1}^{R} g_i^{-1} N_i e^{(\beta-\beta_i)E} e^{(\beta P-\beta_i P_i)\frac{N}{\rho}} e^{F_i}} \\ e^{-F_i} &= \sum_{\{E,\rho,n_{max}\}} P_{ub}(E,\rho,n) e^{(\beta-\beta_i)E} e^{(\beta P - \beta_i P_i)\frac{N}{\rho}} \end{aligned} \tag{24}$$

In the subsequent discussion, we adapt the WHAM equations described above to the specific cases of:

1. Obtaining the free energy barrier as a function of cluster size, $\beta\Delta G(n)$
2. Stitching and reweighted the distribution as a function of E, ρ in order to obtain $\beta\Delta G(\rho)$
3. Constructing the two order parameter free energy $\beta\Delta G(n,\rho)$

Case 1: Stitching free energy as a function of cluster size, $\beta\Delta G(n)$ Here, we obtain the unbiased distribution of all cluster sizes, $P_{ub}(n)$, from which we write the free energy, $\beta\Delta G(n)$. The WHAM equations are used to obtain $P_{ub}(n)$ (at the same temperature and pressure at which the simulations are performed):

$$\begin{aligned} P_{ub}(n) &= \frac{\sum\limits_{i=1}^{R} H_i(n)}{\sum\limits_{i=1}^{R} N_i e^{-F_i}} \\ e^{-F_i} &= \sum_n P_{ub}(n) \end{aligned} \tag{25}$$

Histogram entries $H_i(n)$ are given by:

$$H_i(n) = \frac{1}{\tau_i}\sum_{t=1}^{\tau_i}\begin{cases}\delta(n(t)-n) & \text{if} \quad n_{lo} \le n \le n_{hi} \\ 0 & otherwise\end{cases} \tag{26}$$

While the bias constraints are applied on n_{max}, we track the full cluster size distribution because the approximation $P(n_{max}) \approx P(n)$ does not hold at deep supercooling when the size of the critical

cluster is small[6, 9]. In obtaining the estimates for $H_i(n)$ and consequently for $P_{ub}(n)$ ($\beta\Delta G(n) = -\ln[P_{ub}(n)]$), we only consider data for n values within the bounds $[n^i_{lo}, n^i_{hi}]$ for each simulation, even though all cluster sizes $n \leq n^i_{hi}$ are sampled. This is done because the frequency of occurrence of clusters of size $n_{lo} \leq n \leq n_{hi}$ satisfies the requirement that at least one cluster in the specified size range must be present at any point in time[9]. The normalisation factor for n within the bounds is therefore not meaningfully applicable to values of n outside the bounds; it is therefore simpler to discard data for n outside the bounds.

The stitching using the WHAM procedure can be compared with a procedure where the free energies are stitched by determining the appropriate additive constant, b_d, which minimizes the discrepancy between free energy estimates from different, but overlapping, bias windows, in the overlapping regions. From a set of independent simulations, each indexed by d and having distinct but adjacent bounds for n_{max}, one obtains the free energy differences $\beta\Delta G_d(n)$ up to an undetermined constant, b_d. The constants, b_d, are obtained by minimising the error described in Eq. 27, χ_{HW}, sequentially between overlapping data points from simulations with adjacent bounds.

$$\chi_{HW} = \sum_{d=1}^{N_{sim}} \sum_{n=n^d_{lo}}^{n^d_{hi}} [\beta\Delta G_d(n) - \beta\Delta G_{d+1}(n) - b_d]^2 \tag{27}$$

This is done subject to the constraint

$$\beta\Delta G(0) = 0 \quad \text{if} \quad n^d_{lo} = 0. \tag{28}$$

A comparison of results is shown in Fig. S2 where we find that the two procedures give quantitatively identical results.

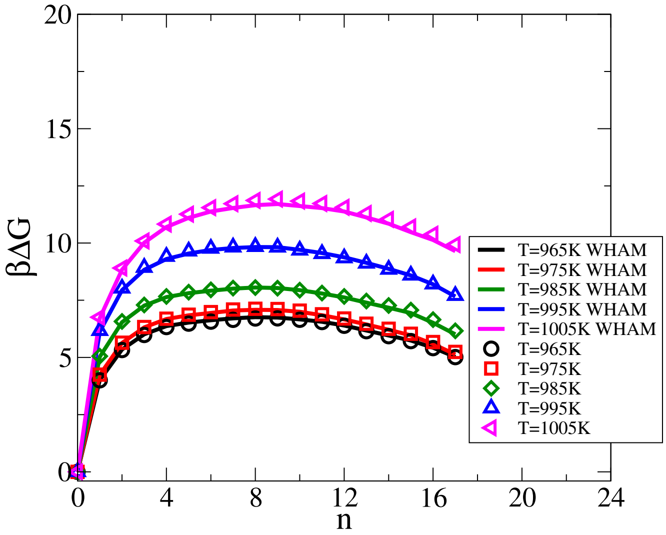

Figure S2: A comparison of free energy stitching procedures using the WHAM equations in Eq. 25 and the minimisation of χ_{HW} in Eq. 27 showing identical results from simulations of $N = 512$ atoms at $P = 0.75\ GPa$.

Case 2: Stitching and reweighting $P_{ub}(E, \rho)$ and obtaining $\beta\Delta G(\rho)$ The following equations are solved self-consistently, with a condition imposed that the largest cluster size, $n_{max} \leq n_{lim}$, when updating the histogram $H_i(E, \rho)$.

$$P_{ub}(E, \rho; \beta, P) = \frac{\sum_{i=1}^{R} g_i^{-1} H_i(E, \rho)}{\sum_{i=1}^{R} g_i^{-1} N_i e^{(\beta-\beta_i)E} e^{(\beta P - \beta_i P_i)N/\rho} e^{F_i}}$$
$$e^{-F_i} = \sum_{\{E,\rho\}} P_{ub}(E, \rho, n) e^{(\beta-\beta_i)E} e^{(\beta P - \beta_i P_i)N/\rho} \tag{29}$$

The rule for updating the histogram $H_i(E, \rho)$ is given by:

$$H_i(E, \rho) = \frac{1}{\tau_i} \sum_{t=1}^{\tau_i} \begin{cases} \delta(E(t) - E)\delta(\rho(t) - \rho) & \text{if} \quad n_{max}(t) \leq n_{lim} \\ 0 & otherwise \end{cases} \tag{30}$$

$P_{ub}(E, \rho; \beta, P)$ is the unbiased sampling probability of energy and density, measured subject to a constraint on n_{max}, weighted on β and P. The density histogram can be obtained by summing over all values of E. The term g_i is the integrated auto-correlation time for each simulation window, shown in Eq. 22. The results from this procedure are shown in Fig. S6, Fig. S5 and Fig. S7.

Case 3: Constructing the two order parameter free energy $\beta\Delta G(n, \rho)$ $\beta\Delta G(n, \rho)$ shows the degree of crystallinity along one axis and the density along the other so that the two liquids can be characterised in relation to the crystalline phase. We write the WHAM equations as in Eq. 20, where we are interested now in $P_{ub}(n, \rho)$. As discussed in the main text and Methods, this is the probability of observing a cluster of size n when the liquid has density, ρ. We begin by noting that the histogram $H(n, \rho)$ is updated as follows:

$$H_i(n, \rho) = \frac{1}{\tau_i} \sum_{t=0}^{\tau_i} \frac{N(n, t)}{N(0, t)} \delta(\rho(t) - \rho) \quad if \quad n_{lo}^i \leq n \leq n_{hi}^i \tag{31}$$

We then write the unbiased probability $P_{ub}(n, \rho)$ as

$$\begin{aligned} P_{ub}(n, \rho; \beta, P) &= \frac{\sum_{i=1}^{R} g_i^{-1} H_i(n, \rho)}{\sum_{i=1}^{R} g_i^{-1} N_i e^{F_i}} \\ e^{-F_i} &= \sum_{\{E, \rho\}} P_{ub}(n, \rho) \end{aligned} \tag{32}$$

The bi-variate distribution, $P(n, \rho)$ yields the full two-order parameter free energy $\beta\Delta G(n, \rho)$.

$$\Delta G(n, \rho) = -k_B T \ln(P(n, \rho)). \tag{33}$$

One may also consider the free energy $\beta\Delta G(n_{max}, \rho)$ however, this would lead to the appearance of an artificial minimum at small n_{max}[14, 15, 9]. Considering $\beta\Delta G(n, \rho)$ allows us to clarify features of the free energy landscape at deep supercooling, as well as to verify the presence of the LDL phase in the absence of any crystalline ordering.

2 Convergence tests for $N = 512$ atoms along the $P = 0.75$ GPa isobar

We measure decorrelation times for key quantities in our umbrella sampling simulations in order to test for the convergence of the free energy estimate, $\beta\Delta G(\rho)$, subject to a constraint on n_{max}. In generating the corresponding autocorrelation functions, we consider two types of time series. The first type is a time series of configurations subject to a given bias potential labelled by ρ_0, the reference density. With this, we compute the autocorrelation of the ρ and Q_6 shown in panels A and B respectively of Fig. S3 and Fig. S4 (**Note:** This time series exhibits discontinuities when a swap occurs). The second type is a time series of each trajectory initialised in a given bias window. These are labelled by the bias potential applied at the initial time and are subjected to different bias potentials over time when parallel tempering swaps are performed. These are continuous trajectories, but the bias potential changes over time, giving a corresponding time series of the reference ρ_0 values. We compute the autocorrelation function of the reference ρ_0, shown in panel C of Fig. S3 and Fig. S4). For the first type of time series, the bias potential remains the same, whereas for the second type, the bias potential changes with time.

In Panel D of the same figures, the mean excursion length away from the initial reference density, or mean return time, is shown for different density windows, indexed ρ_0, subject to the constraint of $n_{max} \leq 4$.

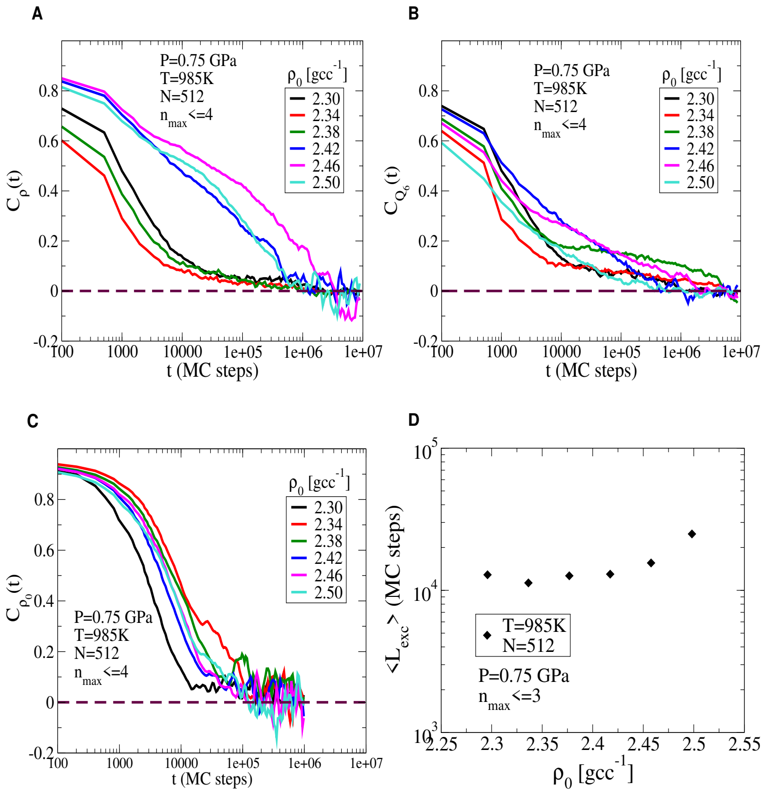

Figure S3: Decay of time auto-correlation function for density (Panel A), Q_6 (Panel B), and for density window index, c_{id}, (Panel C) for each of the density bias windows, ρ_0, subject to the constraint of $n_{max} \leq 4$ at $T = 985K$, $P = 0.75\ GPa$. Panel D shows the mean excursion length or return time as a function of ρ_0 subject to the constraint on n_{max} at $T = 985K$. In each case, the different curves are labelled according to the initial reference density, ρ_0, for the given independent simulation.

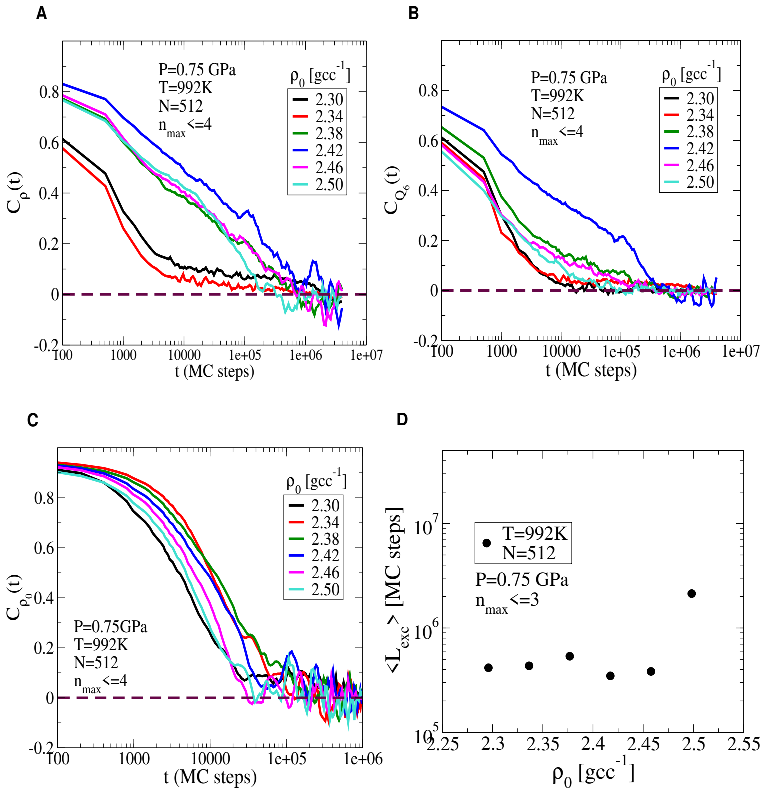

Figure S4: Decay of time auto-correlation function for density (Panel A), Q_6 (Panel B), and for density window index (Panel C) for each of the density bias windows, ρ_0, subject to the constraint of $n_{max} \leq 4$ at $T = 992K$, $P = 0.75\ GPa$. Panel D shows the mean excursion length or return time as a function of ρ_0 subject to the constraint on n_{max}. In each case, the different curves are labelled according to the initial reference density, ρ_0, for the given independent simulation.

Histogram reweighting at $P = 0.75\ GPa$ to test for equilibrium sampling

The histogram reweighting procedure in Eq. 29 is used to obtain the unbiased, reweighted, bivariated distribution $P_{ub}(E, \rho; \beta, P)$ at the target conditions of $T = 1/\beta$, P. The free energy, shown in Fig. S5 and in Fig. S6 is given by $\beta\Delta G(\rho; \beta, P) = -ln(P_{ub}(\rho; \beta, P)$. Note that changing the constraint on n_{max} alters the coexistence temperature at a given isobar (compare Fig. S6 and Fig. S5), however, the feature of coexistence remains and is robust to changes in the choice of the upper bound in n_{max} less than the critical cluster size.

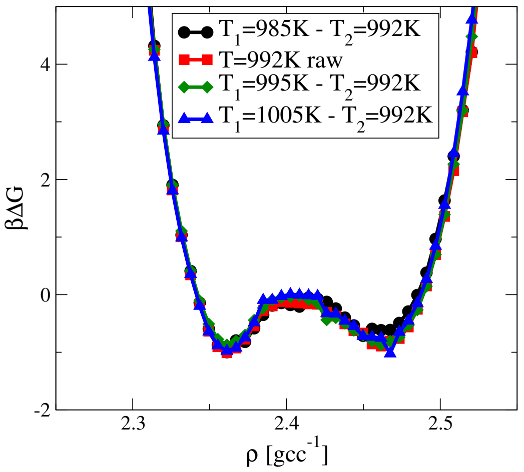

Figure S5: Raw and re-weighted free energies at three temperatures, for $P = 0.75\ GPa$, $N = 512$, with a constraint on n_{max} at $n_{max} \leq 4$. Note that for this constraint, the coexistence temperature is $T = 992K$.

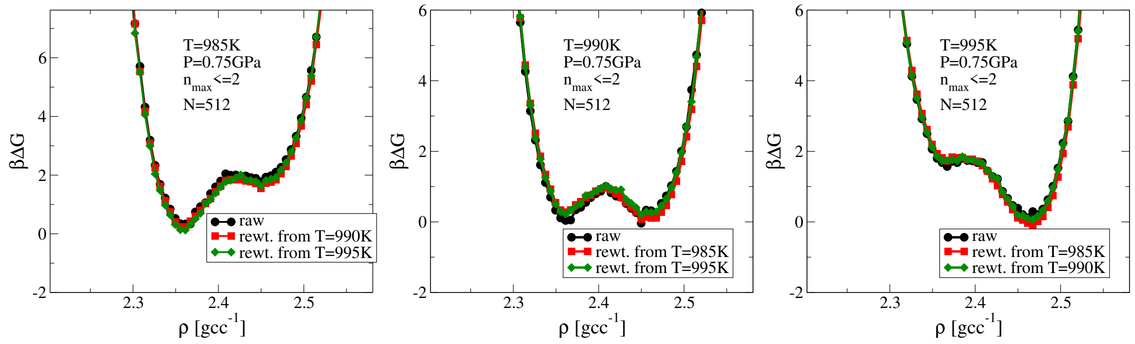

Figure S6: Raw and re-weighted free energies at three temperatures, for $P = 0.75\ GPa$, $N = 512$, with a constraint on n_{max} at $n_{max} \leq 2$. For this constraint of smaller n_{max} (compared to Fig. S5), the coexistence temperature shifts to a lower temperature of $T = 990K$.

Histogram reweighting of $\beta\Delta G(\rho, E)$

In Fig. S7, we show results from applying the histogram reweighting procedure described in Eq. 29 to the bivariate distribution of ρ and the potential energy. By reweighting across temperatures along the $P = 0.75\ GPa$ isobar, we find that directly measured free energy estimates are identical to those obtained by T, P reweighting, which is a strong indication of converged, equilibrium sampling.

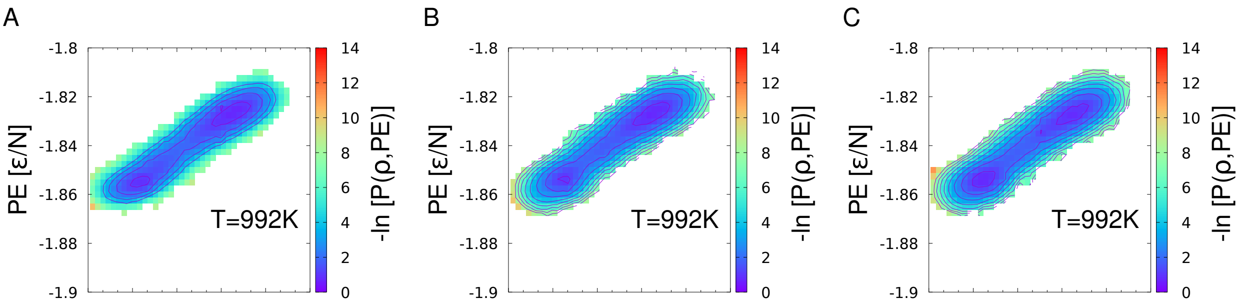

Figure S7: (*Left:*) Re-weighted from $T_1 = 985K$ to $T_2 = 992K$, (*Centre:*) computed directly from umbrella sampling simulations, (*Right:*) re-weighted from $T_1 = 995K$ to $T_2 = 992K$.

3 Comparison of methods far from co-existence

Away from the state points where two liquids co-exist, the two umbrella sampling schemes are expected to give the same results. Close to co-existence, the scheme of only performing parallel tempering swaps across temperature, without constraining the density, may or may not give converged estimates of the free energy on reasonable simulation timescales. This is because the temperature parallel tempering needs to effect a barrier crossing. The comparison is made either side of co-existence along the $P = 0.75\ GPa$ isobar.

3.1 Convergence and sampling tests for umbrella sampling runs constraining n_{max} only

The decay of the auto-correlation functions for ρ, Q_6 from the time series of configurations simulated at a given temperature are shown in Fig. S8, Fig. S9, Fig. S10 and Fig. S11, panels A and B. The temperatures chosen are those outside the co-existence region of LDL and HDL. We also construct a time series of trajectories initialised at a given temperature, T, where each trajectory is subject to different temperatures over time as parallel tempering swaps are performed. This time series is then used to construct the autocorrelation function, $C_T(t)$, shown in panel C of Fig. S8, Fig. S9, Fig. S10 and Fig. S11.

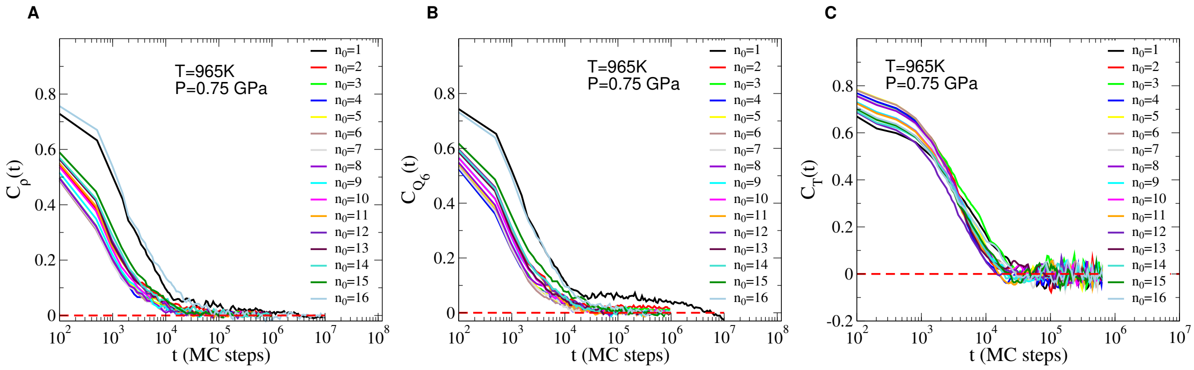

Figure S8: Autocorrelation of density, $C_\rho(t)$ (Panel A), Q_6 , $C_{Q_6}(t)$ (Panel B) and simulation temperature $C_T(t)$ (Panel C) at $T = 965K$, $P = 0.75\ GPa$ with $N = 512$. The initial temperature for each simulation is as shown in the panel, the different curves are labelled according to the midpoint of the n_{max} bias window, n_0.

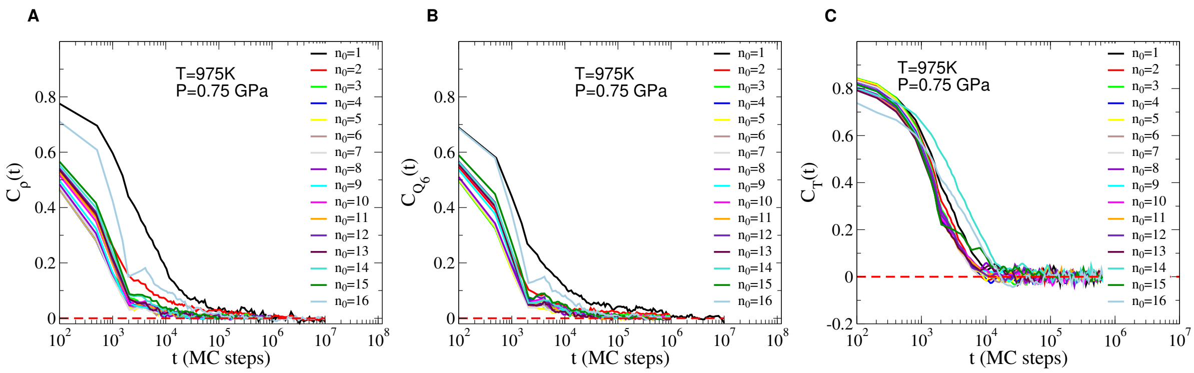

Figure S9: Autocorrelation of density, $C_\rho(t)$ (Panel A), Q_6 , $C_{Q_6}(t)$ (Panel B) and simulation temperature $C_T(t)$ (Panel C) at $T = 975K$, $P = 0.75\ GPa$ with $N = 512$. The initial temperature for each simulation is as shown in the panel, the different curves are labelled according to the midpoint of the n_{max} bias window, n_0.

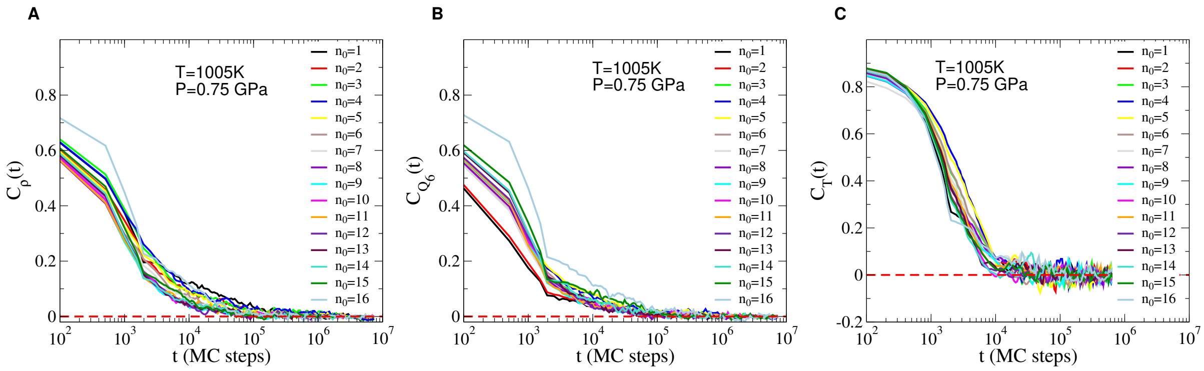

Figure S10: Autocorrelation of density, $C_\rho(t)$ (Panel A), Q_6 , $C_{Q_6}(t)$ (Panel B) and simulation temperature $C_T(t)$ (Panel C) at $T = 1005K$, $P = 0.75\ GPa$ with $N = 512$. The initial temperature for each simulation is as shown in the panel, the different curves are labelled according to the midpoint of the n_{max} bias window, n_0.

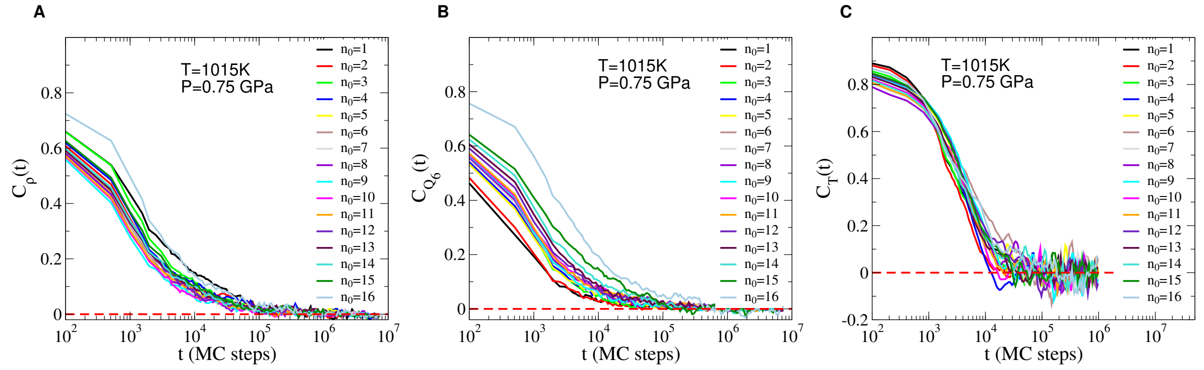

Figure S11: Autocorrelation of density, $C_\rho(t)$ (Panel A), Q_6 , $C_{Q_6}(t)$ (Panel B) and simulation temperature $C_T(t)$ (Panel C) at $T = 1005K$, $P = 0.75\ GPa$ with $N = 512$. The initial temperature for each simulation is as shown in the panel, the different curves are labelled according to the midpoint of the n_{max} bias window, n_0.

Results from full two-order parameter umbrella sampling, constraining n_{max} and ρ, are compared with results from simulations where only n_{max} is constrained. In the latter case, parallel tempering across temperatures enhances sampling of density. This procedure does not work close to LDL-HDL co-existence conditions since swaps across the density range occur infrequently, affecting estimates of barrier height and basin depth. Away from co-existence conditions, the two methods give the same results, as shown in Fig. S12.

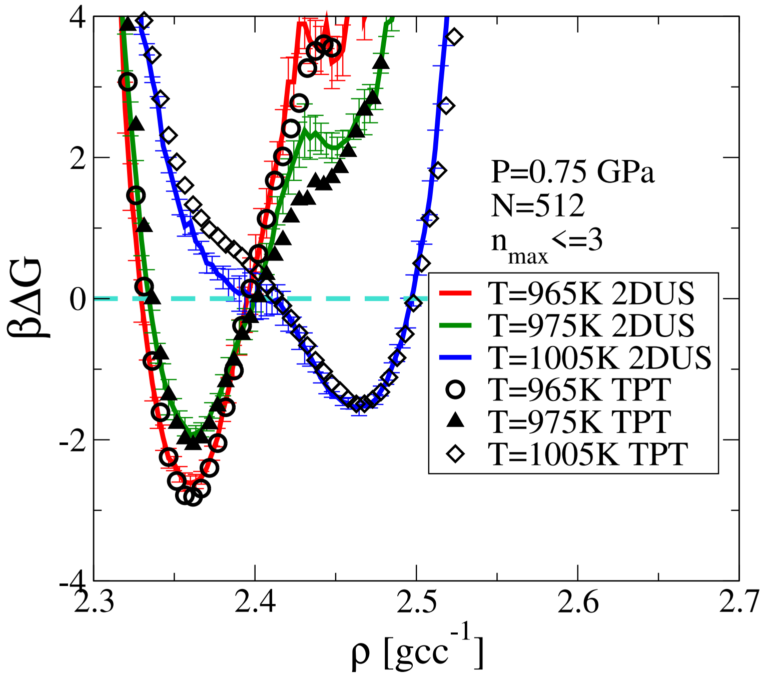

Figure S12: Comparison of free energy along density, ρ, with two order parameter umbrella sampling (solid symbols) and one order parameter umbrella sampling along n_{max} and enhanced sampling of density with parallel tempering across temperatures (hollow black symbols). For the two order parameter US simulations, sampling of different densities is enhanced by including a bias potential along ρ for 6 different ρ_0 values. A comparison is made for the density distribution subject to the constraint on n_{max} to test for consistency across methods. Free energy is obtained for the unbiased distributions for simulations of $N = 512$ atoms. Convergence is tested by monitoring the distribution of residence times resulting from parallel tempering and from monitoring the decay of the auto-correlation functions for Q_6 and ρ.

4 Free energy at $P = 0GPa$ and $P = 1.5GPa$

We perform a similar investigation along two other isobars in the sub-critical regime, finding that the liquid-liquid phase transition occurs at the expected state points in each case. Along one super-critical isobar, we find no evidence of a discontinuous change in the nature of the liquid. This is expected since the two liquids are expected to be indistinguishable in the supercritical regime. Fig. S13 and Fig. S14 show the autocorrelation functions and mean parallel tempering excursion lengths at state points close to co-existence for $P = 0$ GPa and $P = 1.5$ GPa respectively.

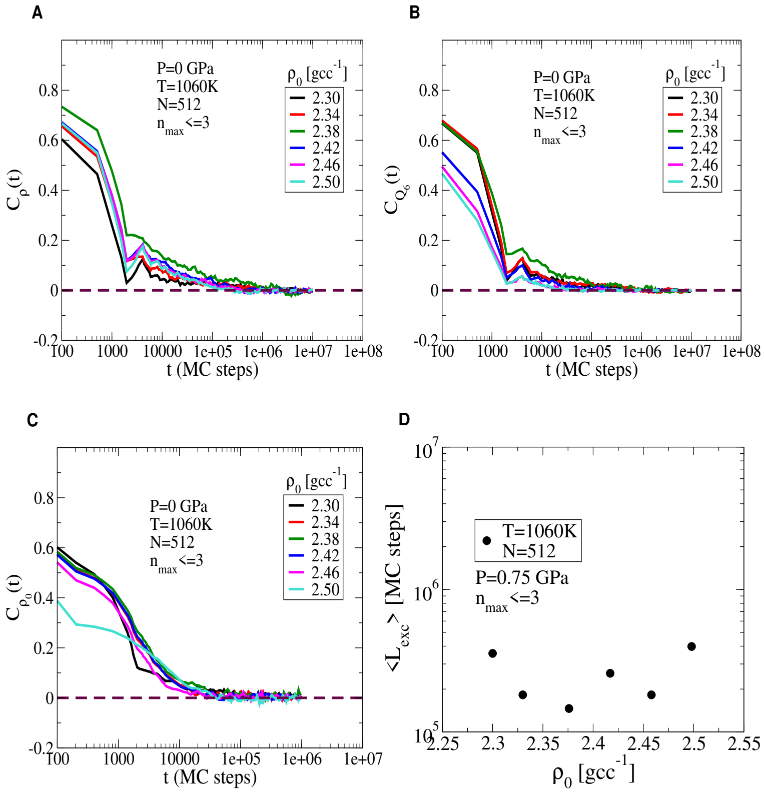

Figure S13: Decay of time auto-correlation function for density (Panel A), Q_6 (Panel B), and for density window index (Panel C) for each of the density bias windows, ρ_0, subject to the constraint of $n_{max} \leq 3$ at $T = 1060K$, $P = 0$ GPa. Panel D shows the mean excursion length or return time as a function of ρ_0 subject to the constraint on n_{max}. In each case, the different curves are labelled according to the initial reference density, ρ_0, for the given independent simulation.

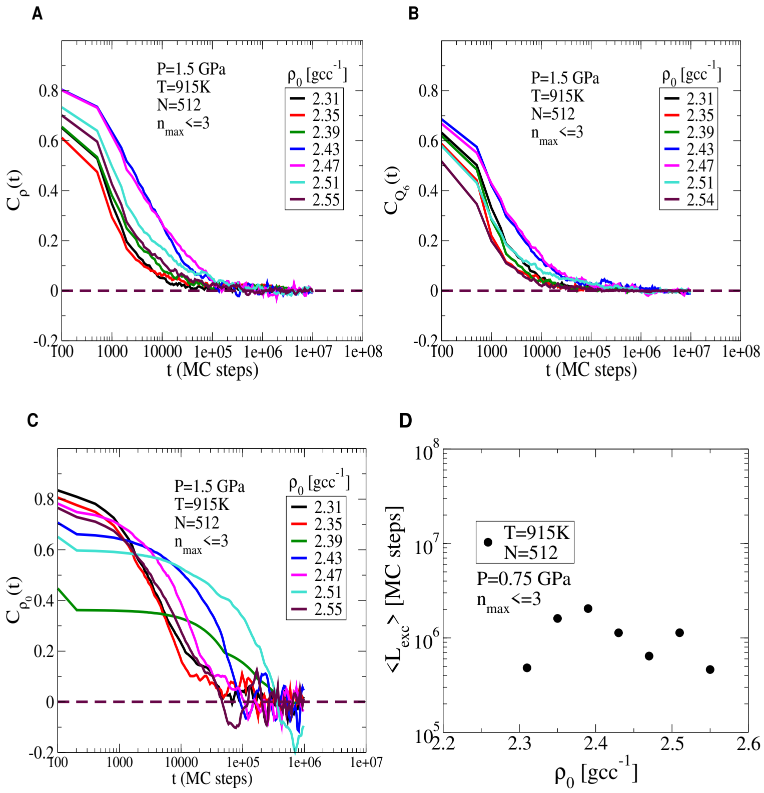

Figure S14: Decay of time auto-correlation function for density (Panel A), Q_6 (Panel B), and for density window index (Panel C) for each of the density bias windows, ρ_0, subject to the constraint of $n_{max} \leq 3$ at $T = 915K$, $P = 1.5\ GPa$. Panel D shows the mean excursion length or return time as a function of ρ_0 subject to the constraint on n_{max}. In each case, the different curves are labelled according to the initial reference density, ρ_0, for the given independent simulation.

Co-existence conditions are also identified along other isobars in Fig. S15 and Fig. S16. $\beta\Delta G(\rho)$ is shown, subject to constraint on n_{max}, demonstrating the shift in the typical density of the liquid from high to low temperature and the region where the distributions are bi-modal. Results are shown along the $P = 0\ GPa$ isobar in Fig. S15 and along the $P = 1.5\ GPa$ isobar in Fig. S16.

5 Free energy reconstructions at larger system sizes

In Fig. 2 main manuscript, we show the free energy profile as a function of density along the $P = 0.75\ GPa$ isobar at four system sizes, $N = 512$, $N = 800$, $N = 1000$ and $N = 2000$. For the case where the two liquids can have a stable interface between them, the barrier height between the two liquids is expected to scale as $N^{2/3}$, which is shown in Fig. 2 in the main text. The low density phase is a disordered phase, as shown by checking the scaling of Q_6 in the low density basin with N, which goes as $N^{-1/2}$ as shown in Fig. S20. This is indicative of a disordered phase, whereas ordered phases would have constant Q_6 for all N. The sections that follow show the autocorrelation functions and parallel tempering statistics for the corresponding sets of simulations at larger system sizes. Convergence of simulations for $N = 800$ near coexistence conditions are shown in Fig. S17, for $N = 1000$ in Fig. S18, and for $N = 2000$ in Fig. S19.

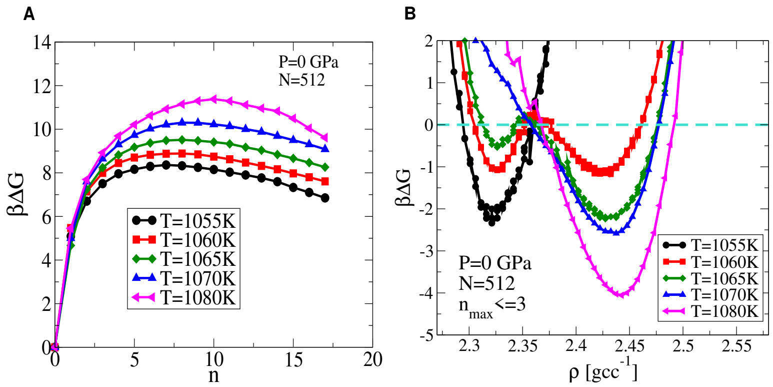

Figure S15: Free energy barrier to crystallisation along the $P = 0\ GPa$ isobar (Panel A) and free energy as a function of density along the $P = 0\ GPa$ isobar (Panel B). The free energy along density is obtained from the unweighted density distributions measured subject to constraint on n_{max}.

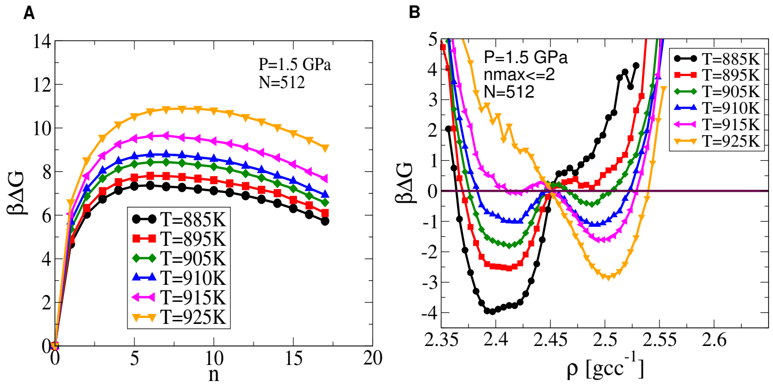

Figure S16: Free energy barrier to crystallisation along the $P = 1.5\ GPa$ isobar (Panel A) and free energy as a function of density along the $P = 1.5\ GPa$ isobar (Panel B). The free energy along density is obtained from the unweighted density distributions measured subject to constraint on n_{max}.

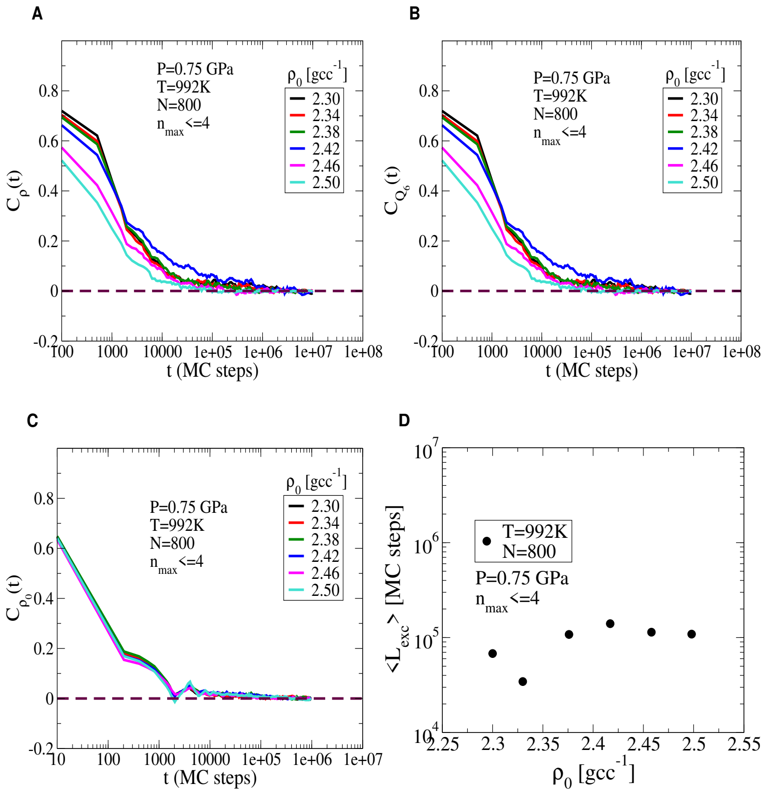

Figure S17: Decay of time auto-correlation function for density (Panel A), Q_6 (Panel B), and for density window index (Panel C) for each of the density bias windows, ρ_0, subject to the constraint of $n_{max} \leq 4$ at $T = 992K$, $P = 0.75\ GPa$. Panel D shows the mean excursion length or return time as a function of ρ_0 subject to the constraint on n_{max}. In each case, the different curves are labelled according to the initial reference density, ρ_0, for the given independent simulation.

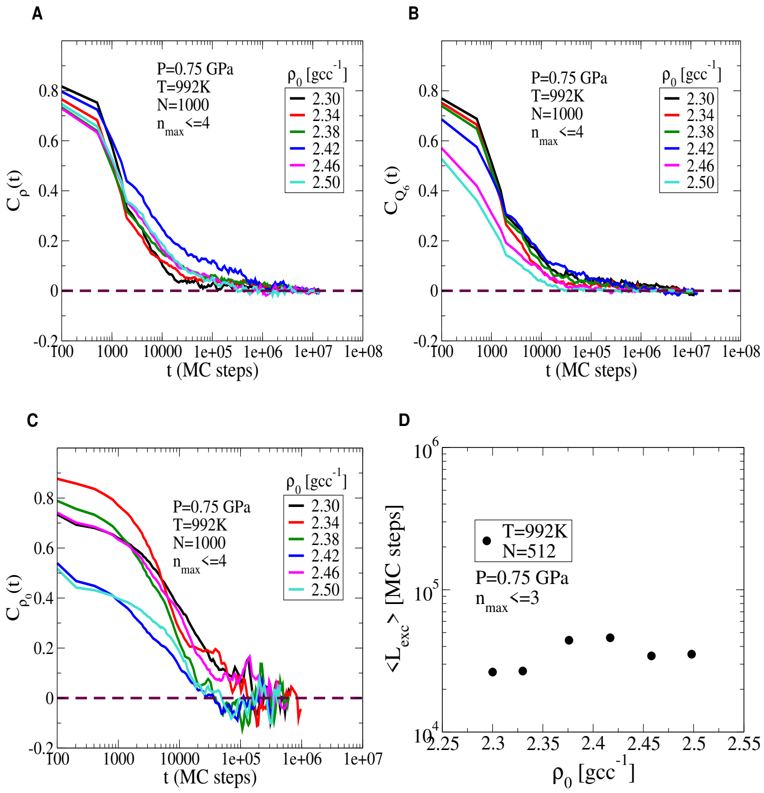

Figure S18: Decay of time auto-correlation function for density (Panel A), Q_6 (Panel B), and for density window index (Panel C) for each of the density bias windows, ρ_0, subject to the constraint of $n_{max} \leq 4$ at $T = 992K$, $P = 0.75\ GPa$. Panel D shows the mean excursion length or return time as a function of ρ_0 subject to the constraint on n_{max}. In each case, the different curves are labelled according to the initial reference density, ρ_0, for the given independent simulation.

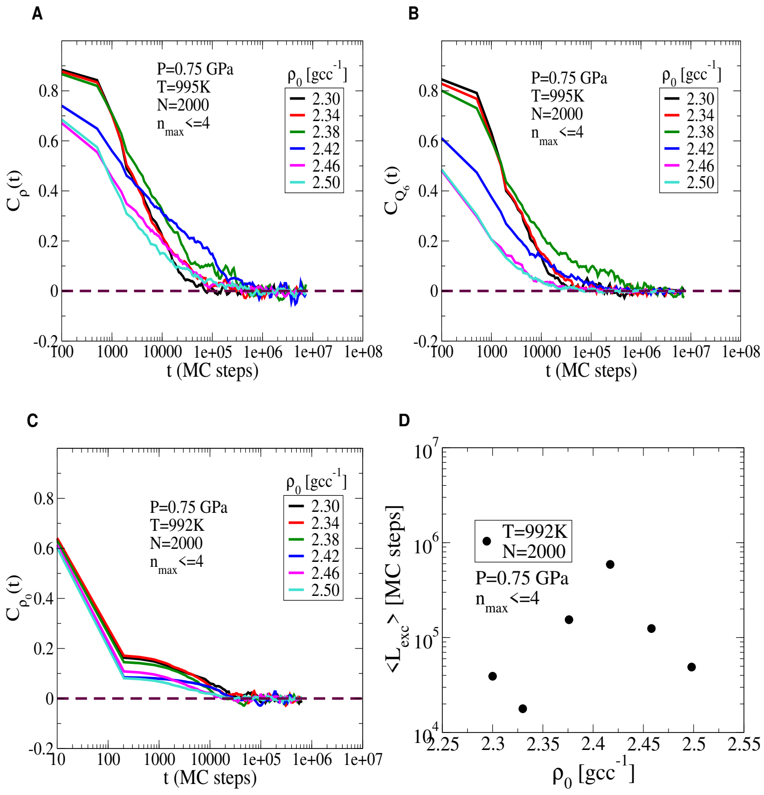

Figure S19: Decay of time auto-correlation function for density (Panel A), Q_6 (Panel B), and for density window index (Panel C) for each of the density bias windows, ρ_0, subject to the constraint of $n_{max} \leq 3$ at $T = 992K$, $P = 0.75\ GPa$. Panel D shows the mean excursion length or return time as a function of ρ_0 subject to the constraint on n_{max}. In each case, the different curves are labelled according to the initial reference density, ρ_0, for the given independent simulation.

Scaling of LDL basin depth with system size

Fig. S20 shows the decrease in basin depth as system size is increased, at $P = 0.75\ GPa,\ T = 985K$. The average Q_6 subject to constraint on ρ and n_{max} is shown to scale as $N^{-1/2}$ in the main manuscript Fig. 2.

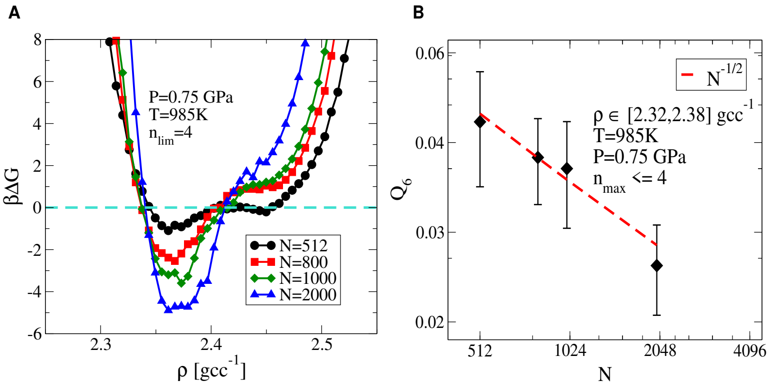

Figure S20: $\beta\Delta G(\rho)$ at $P = 0.75$ GPa and $T = 985K$ from simulations and reweighting at 4 system sizes, $N = 512,\ 800,\ 1000,\ 2000$ (panel A). These conditions correspond to a single stable LDL phase. The scaling of the Q_6, measured subject to constraint on ρ, is shown in panel B.

6 Fit to the Ising Universality class

The analysis of the critical fluctuations of the order parameter is carried out by comparing the distribution of the order parameter, $M = \rho + sE$ with the reference distribution:

$$P_{ising}(M) \propto exp\left\{-\left(\frac{M^2}{M_0^2} - 1\right)^2 \left(a\frac{M^2}{M_0^2} + c\right)^2\right\} \tag{34}$$

For $M_0 = 1.1341665$, $a = 0.158$, $c = 0.776$, one obtains a universal distribution of unit variance. By identifying the parameter for the LLPT, $r = \rho + sE$, and shifting and scaling it as $M = \frac{r - \langle r \rangle}{\sigma_r}$, one obtains a distribution of unit variance, which is thus system-size independent. We compare the difference between the distributions, $P(M)$, and $P_{ising}(M)$, and identify the set of T_c, P_c, s that minimise the difference between them. The minimisation is performed using an implementation of the Nelder-Mead optimisation scheme available with the SciPy optimization library[16]. The critical point can be estimated using this optimisation procedure as the T_c, P_c for which the distribution best matches the reference distribution. In Fig. S21, we show the bivariate free energy as a function of ρ and the potential energy, E, at the identified critical point conditions. Histogram data is reweighted from the $P = 0\ GPa$ isobar at different temperatures to produce estimates in the vicinity of the critical point.

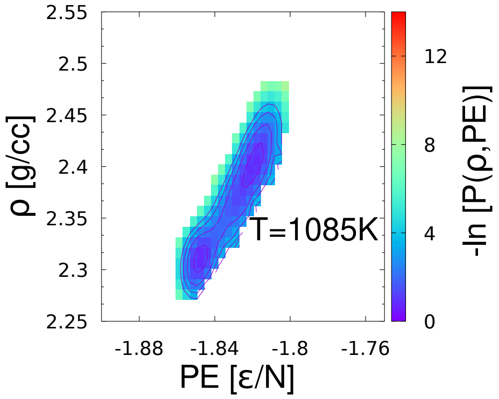

Figure S21: Bivariate free energy as a function of ρ and the potential energy, E, at the identified T_c, P_c of $T = 1085.5K,\ P = -0.5\ GPa$ showing the weakly double well feature characteristic of critical fluctuations.

References

[1] Frank H Stillinger and Thomas A Weber. Computer simulation of local order in condensed phases of silicon. *Physical review B*, 31(8):5262, 1985.

[2] Paul J Steinhardt, David R Nelson, and Marco Ronchetti. Bond-orientational order in liquids and glasses. *Physical Review B*, 28(2):784, 1983.

[3] Vishwas V Vasisht, John Mathew, Shiladitya Sengupta, and Srikanth Sastry. Nesting of thermodynamic, structural, and dynamic anomalies in liquid silicon. *The Journal of chemical physics*, 141(12):124501, 2014.

[4] JS Van Duijneveldt and D Frenkel. Computer simulation study of free energy barriers in crystal nucleation. *The Journal of chemical physics*, 96(6):4655–4668, 1992.

[5] Pieter Rein Ten Wolde, Maria J Ruiz-Montero, and Daan Frenkel. Numerical evidence for bcc ordering at the surface of a critical fcc nucleus. *Physical review letters*, 75(14):2714, 1995.

[6] Pieter-Reináten Wolde et al. Simulation of homogeneous crystal nucleation close to coexistence. *Faraday discussions*, 104:93–110, 1996.

[7] Flavio Romano, Eduardo Sanz, and Francesco Sciortino. Crystallization of tetrahedral patchy particles in silico. *The Journal of chemical physics*, 134(17):174502, 2011.

[8] Tobias A Kesselring, Erik Lascaris, Giancarlo Franzese, Sergey V Buldyrev, Hans J Herrmann, and H Eugene Stanley. Finite-size scaling investigation of the liquid-liquid critical point in st2 water and its stability with respect to crystallization. *The Journal of Chemical Physics*, 138(24):244506, 2013.

[9] Yagyik Goswami, Vishwas V Vasisht, Daan Frenkel, Pablo G Debenedetti, and Srikanth Sastry. Thermodynamics and kinetics of crystallization in deeply supercooled stillinger–weber silicon. *The Journal of Chemical Physics*, 155(19):194502, 2021.

[10] Francesco Ricci, Jeremy C Palmer, Yagyik Goswami, Srikanth Sastry, C Austen Angell, and Pablo G Debenedetti. A computational investigation of the thermodynamics of the stillinger-weber family of models at supercooled conditions. *Molecular Physics*, pages 1–15, 2019.

[11] Shankar Kumar, John M Rosenberg, Djamal Bouzida, Robert H Swendsen, and Peter A Kollman. The weighted histogram analysis method for free-energy calculations on biomolecules. i. the method. *Journal of computational chemistry*, 13(8):1011–1021, 1992.

[12] John D Chodera, William C Swope, Jed W Pitera, Chaok Seok, and Ken A Dill. Use of the weighted histogram analysis method for the analysis of simulated and parallel tempering simulations. *Journal of Chemical Theory and Computation*, 3(1):26–41, 2007.

[13] Pablo G Debenedetti, Francesco Sciortino, and Gül H Zerze. Second critical point in two realistic models of water. *Science*, 369(6501):289–292, 2020.

[14] Lutz Maibaum. Comment on "elucidating the mechanism of nucleation near the gas-liquid spinodal". *Physical review letters*, 101(1):019601, 2008.

[15] Suman Chakrabarty, Mantu Santra, and Biman Bagchi. Chakrabarty, santra, and bagchi reply. *Physical Review Letters*, 101(1):019602, 2008.

[16] Fuchang Gao and Lixing Han. Implementing the nelder-mead simplex algorithm with adaptive parameters. *Computational Optimization and Applications*, 51(1):259–277, 2012.
